# Quantifying the Relative Importance of Phylogeny and Environmental Preferences As Drivers of Gene Content in Prokaryotic Microorganisms

**DOI:** 10.3389/fmicb.2016.00433

**Published:** 2016-03-31

**Authors:** Javier Tamames, Pablo D. Sánchez, Pablo I. Nikel, Carlos Pedrós-Alió

**Affiliations:** ^1^Departamento de Biología de Sistemas, Centro Nacional de Biotecnología, Consejo Superior de Investigaciones CientíficasMadrid, Spain; ^2^Departament de Biologia Marina i Oceanografia, Institut de Ciències del Mar, Consejo Superior de Investigaciones CientíficasBarcelona, Spain

**Keywords:** habitat preference, phylogenetic diversity, genome evolution, genome content, environmental preference, bioinformatics

## Abstract

Two complementary forces shape microbial genomes: vertical inheritance of genes by phylogenetic descent, and acquisition of new genes related to adaptation to particular habitats and lifestyles. Quantification of the relative importance of each driving force proved difficult. We determined the contribution of each factor, and identified particular genes or biochemical/cellular processes linked to environmental preferences (i.e., propensity of a taxon to live in particular habitats). Three types of data were confronted: (i) complete genomes, which provide gene content of different taxa; (ii) phylogenetic information, *via* alignment of 16S rRNA sequences, which allowed determination of the distance between taxa, and (iii) distribution of species in environments *via* 16S rRNA sampling experiments, reflecting environmental preferences of different taxa. The combination of these three datasets made it possible to describe and quantify the relationships among them. We found that, although phylogenetic descent was responsible for shaping most genomes, a discernible part of the latter was correlated to environmental adaptations. Particular families of genes were identified as environmental markers, as supported by direct studies such as metagenomic sequencing. These genes are likely important for adaptation of bacteria to particular conditions or habitats, such as carbohydrate or glycan metabolism genes being linked to host-associated environments.

## Introduction

Microorganisms inherit a genome from their parent cells that reflects their phylogeny. The genes within the genome determine the functions the organism can carry out and, thus, the places where it can live. At the same time, microbes are confronted with a variety of habitats that impose particular constraints on them. Indeed, environments can be classified on this basis (Tamames et al., 2010). Particular genes are required by bacteria in order to overcome the hurdles imposed by such environmental constraints. The interplay between these two forces, related to evolution and ecology respectively, is the cause of the observed distribution of microbial taxa in different environments, and it has also resulted in the partitioning of genomes into a core and a flexible part (Mira et al., 2010). The core genome includes all the essential genes for reproduction and is mostly inherited vertically (Collins and Higgs, 2012). This core set of genes reflects phylogeny rather strictly, although some disrupting events of lateral gene transfer have been proposed for them (Wolf et al., 1999; Acinas et al., 2004). The flexible genome includes genes necessary to adapt to different environments, such as nutrient acquisition or adhesion to particles. If adaptation did not exist, every taxon would be able to live in one niche only, predetermined by its inherited genome. If, on the other hand, adaptation were limitless, microbes would be more related to those co-existing in the same habitats than to their phylogenetic relatives.The reality is obviously somewhere in the middle and microbiologists have wondered about the relative importance of these two forces for a long time (von Mering et al., 2007; Philippot et al., 2010; Tamames et al., 2010; Martiny et al., 2013). The question is relevant to determine whether bacterial taxa have ecological coherence (Philippot et al., 2010). This has been implicitly assumed in many microbial ecology studies, when ecological traits are assigned to whole bacterial classes. For example, β-proteobacteria usually inhabit freshwaters and not marine waters (Kirchman et al., 2005), and marine Bacteroidetes are considered to be decomposers of particulate organic matter rather than dissolved organic matter (Fernández-Gómez et al., 2013). The question can also be examined from the opposite point of view: how much ecological diversity can be found within a lineage?

If diversity is large, the lineage could be distributed in many environments and some of its members could be cosmopolitan. On the other hand, if the ecological diversity of lineages is low, they should be restricted to a few environments. Lineages should have habitat preferences. Indeed, we showed in a previous work that even though prokaryotic taxa are remarkably cosmopolitan (Tamames et al., 2010), in many instances they show environmental preferences that shape the diversity found in different environments.

It is reasonable to expect that habitat preferences will have a reflection on the genomes of the prokaryotic organisms. For instance, the presence of oxygen or alternative electron acceptors imposes aerobic/anaerobic metabolism, which translates into different modes of operation in the respiratory chain, oxygen in the first case and panoply of organic and inorganic compounds in the second. The presence of light allows the existence of phototrophic metabolisms and the synthesis of organic matter via photosynthesis. Carbon sources also determine autotrophic/heterotrophic metabolism, the latter capable of using many different and alternative substrates for growth. Guilds of bacteria having different metabolisms can be linked together producing metabolic interactions, either competitive or cooperative (Freilich et al., 2011; Foster and Bell, 2012; Pascual-García et al., 2014). All these metabolic alternatives require particular sets of genes and, therefore, gene content is expected to be related to environmental preferences. To what extent is this relationship determinant of environmental adaptations (i.e., how large is the genetic rearrangement leading to these adaptations) is unclear.

On the other hand, it may seem obvious that the genomic content is largely determined by phylogenetic proximity: close species tend to have similar genomes (Snel et al., 1999; Konstantinidis and Tiedje, 2005; Zaneveld et al., 2010). Therefore, gene content is modulated by these two contributions: phylogenetic proximity and adaptation due to environmental preferences. The relationship between gene content and the environment remains to be clarified. For instance, it is not clear whether there are any cases of closely related taxa having different gene content in response to environmental adaptations. Or, on the contrary, whether there are cases of evolutionary convergence so that phylogenetically distant taxa may have similar genomes because they live in similar environments.

In summary, the interplay between gene content, environmental preferences and phylogenetic proximity (or divergence) can be rather flexible. In the present work we aimed to quantify such relationships through the comparison of genera from the genetic (particularly metabolic), ecological, and phylogenetic points of view. This quantification, plus the determination of a set of genes linked to environmental preferences, can be relevant for applied microbial physiology and synthetic biology. For instance, some of these marker genes may prove useful for metabolic engineering strategies leading to alter the habitat range of particular species.

## Materials and methods

A brief explanation on the procedure for deriving the different datasets used in this work is provided below. The fully detailed method is shown in Figure S1 in the Supplementary Material.

### Gene content matrix (Figure S1A)

A set of 1384 completely sequenced prokaryotic genomes was used as the source of gene content information. Clusters of orthologous groups (COGs; Tatusov et al., 1997) were used as a source of functional annotations for genomes. To obtain a set of genomes annotated with comparable completeness, we analyzed the distribution of the number of COGs vs. genomic size in the 1384 genomes. A direct relationship was found between these two variables for most genomes (Figure S2 in the Supplementary Material), and we removed 98 genomes that did not conform to the general trend, obtaining a final set of 1286 genomes belonging to 992 different species. An initial gene content matrix was derived, with species in rows and the abundance of the 4873 COGs in each species as columns. For species with several strains, we averaged the abundance of each COG across all the strains.

Genus was chosen as the working rank because the assignment of environmental sequences to species could not be resolved in many instances, and because many species have been observed rarely in natural samples. Mapping species to genera both facilitates the classification and reduces the number of taxonomic units to work with. To generate a gene content matrix at the genus level, the abundance of COGs for all the species belonging to each genus was averaged. Thus, we obtained a gene content matrix of 4873 COGs in 503 genera. We were able to generate also sub-matrices for particular subsets of genes, like those belonging to particular metabolic pathways or functional categories, simply by selecting the COGs involved in such processes. We also recorded several phenotypic (acidophilic, halophilic, psychrophilic, termophilic, alkalophilic) and metabolic characteristics (phototrophic, nitrate reducer, sulfate reducer, methanogen, and reduced, streamlined genomes) for the taxa in this study, according to the literature.

### Environmental preferences matrix (Figure S1B)

- Assignment of samples to environments. We retrieved 2,310,674 16S rDNA sequences corresponding to 15,642 samples, as deposited in GenBank ENV section and collected in the envDB database (http://botero.cnb.csic.es/envDB; Pignatelli et al., 2009) as of December 2013. Out of the 15,642 samples, 12,384 were classified in the envDB environmental categories, slightly modified regarding the original classification (Pignatelli et al., 2009).
- Assignment of environmental sequences to operational taxonomic units (OTUs). The 16S rDNA sequences were clustered into OTUs across all samples. The 16S rDNA sequences were clustered using the program CD-HIT (Li and Godzik, 2006) into operational taxonomic units, sharing at least 98% identity and aligning along 80% of their lengths. This generated 608,456 OTUs. Taxonomic assignment of the OTUs was done as a consensus of RDP classifier results and a BLASTN homology search using the GreenGenes database (DeSantis et al., 2006), looking for the taxonomic coherence of the best hits. In this way, we classified 201,078 OTUs to genera.
- Construction of the environmental preference matrix. We used the environmental classification of the samples, the distribution of OTUs in samples, and the taxonomic assignment of OTUs to produce an environmental matrix composed of the abundance of each taxon in each environment (Tamames et al., 2010). This matrix of environmental abundance contained the number of samples belonging to each environment in which at least one representative of the given genus had been found.

Then we obtained a measure of association between each genus and each environment. We named this parameter “affinity.” A two-tailed Fisher's exact test was used, calculating a *p*-value for the significance of such associations that was corrected by False Discovery Rate (FDR; Benjamini et al., 2001). In this way we obtained a matrix of environmental preferences composed of one environmental vector for each genus, containing the affinity for different environments.

### Distance and correlation matrices

Each pair of genera was compared using their genetic and environmental similarities, and phylogenetic distances. For doing this, we quantified each of these magnitudes as indicated below.

- Phylogenetic distance (Figure S1C). We used 16S rRNA sequences from the GreenGenes database (DeSantis et al., 2006) to obtain estimates of the phylogenetic distances between genera. First, we selected a representative full-length 16S sequence for each prokaryotic species in the database, usually the type strain. Then, we calculated the distance between the aligned sequences as substitutions per position using RaxML with a GTRGAMMA model (Stamatakis, 2014). Distances between genera were calculated as the median of the distances between the species belonging to those genera. The full set of distances between taxa produced a phylogenetic distance matrix (d_phylo_).
- Gene content correlation (Figure S1D). Using the data in the gene content matrix, we generated a gene content correlation matrix (c_gen_) between genera by means of the Spearman's correlation between their gene content vectors (see Figure S1B). Higher correlations indicate more similar gene content.
- Environmental correlation (Figure S1D). The environmental correlations between two genera (c_env_) were calculated also by the Spearman's coefficients between their environmental vectors.
- Co-occurrence strength (Figure S1D). We recorded the number of samples in which two genera co-occurred. A measure of the likelihood of the association was calculated as the *p*-value of a Fisher's exact test (FDR corrected) of their co-occurrence value. This value is not dependent on the environmental classification used. We refer to the −log(*p*-value) as the co-occurrence strength (s_cooc_). Higher s_cooc_ values indicate stronger association.
- Combination of matrices (Figure S1E). All generated matrices: d_phylo_,c_gen_,c_env_, and s_cooc_ were combined in one single matrix that contained the appropriate values for each combination of two genera

### Comparison of magnitudes

To analyze the relationships between d_phylo_,c_gen_,c_env_, and s_cooc_, we used box-plots for all combinations of two distances. The box-plot shows the distribution of values of the variable plotted in the *y*-axis for discrete values of the variable in the *x*-axis. Figure S3 in the Supplementary Material shows an example of the way in which this was done. Each box-plot shows how the variable in the *y*-axis responds to the changes of the variable in the *x*-axis. Mantel tests (Mantel and Valand, 1970) were used to evaluate the correlation between genomic (c_gen_) and either phylogenetic (d_phylo_), environmental (c_env_) or co-occurrence (s_cooc_) matrices. Partial Mantel tests were used to calculate partial correlations between two matrices controlling for effects of a third matrix.

## Results

**Figure 1:**
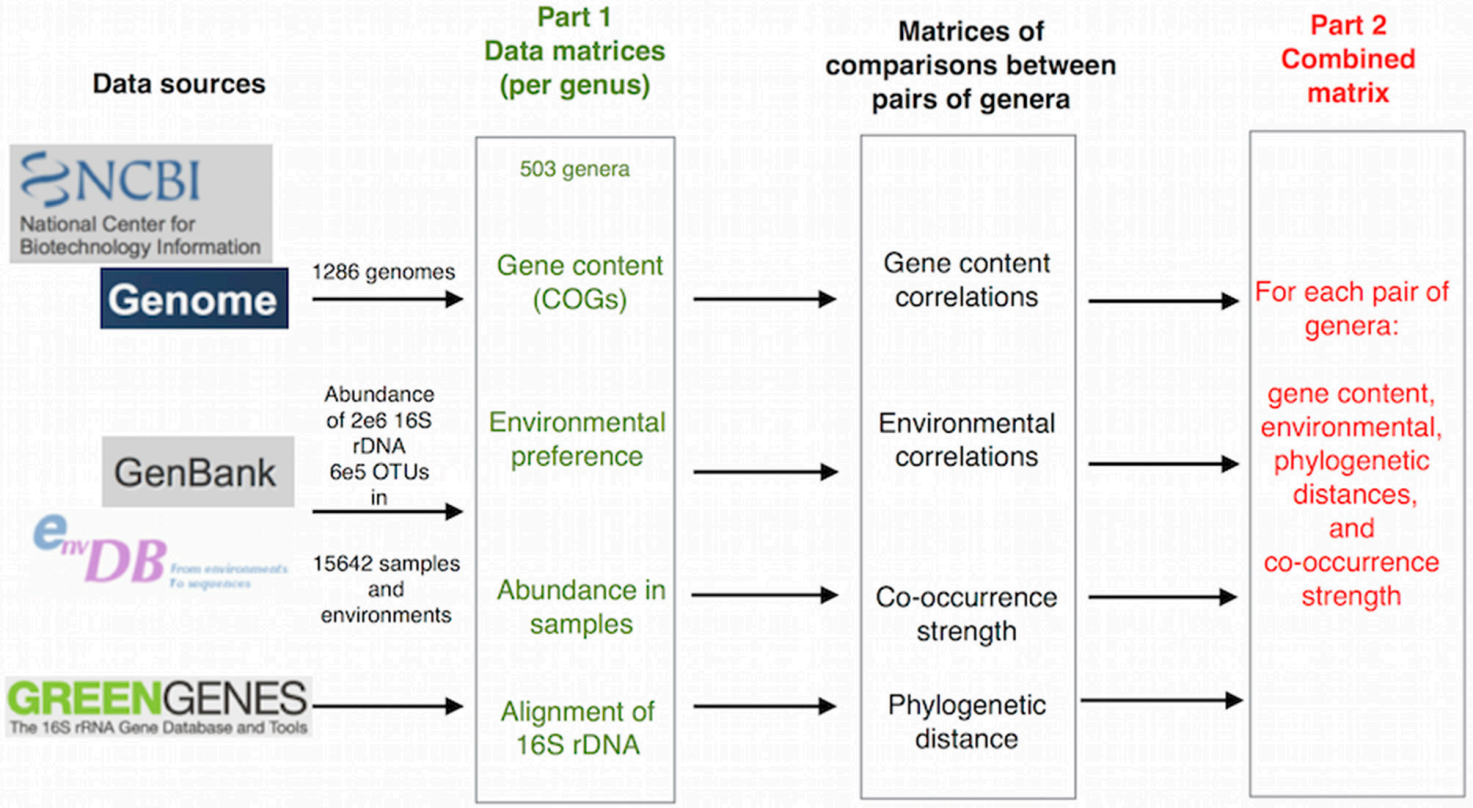
**Outline of the procedure followed**. Primary data were taken from NCBI genomes, GenBank Env and Greengenes. Matrices of properties for every genus were created with these data. Phylogenetic distances between genera were obtained from GeenGenes alignment. Correlations between each pair of genera were computed to generate gene content and environmental correlations, and co-occurrence strength was calculated by a Fisher test of the co-occurrence data. Finally, a combined matrix was created with pairs of genera in rows and the four measures in four columns. The matrices shown in green were used in the first part of the paper and the combined matrix in red in the second. A more detailed description can be found in Figure S1 in the Supplementary Material.

We followed two approaches for the analysis, using two different but linked types of data: (1) matrices of gene content and environmental preferences for each genus were built to explore the relationships between these two characteristics. Gene content information was taken from the analysis of completely sequenced genomes, and environmental preferences were calculated from the frequency of observation of each genus in environmental samples of 16S rDNA sequences (Pignatelli et al., 2009), and (2) comparisons of gene content, environmental preferences, co-occurrence and phylogenetic relationship between pairs of genera were used to provide a quantification of their reciprocal influences (Figure 1]).

### Part 1. relationships between gene content and environmental preferences

**Figure 2:**
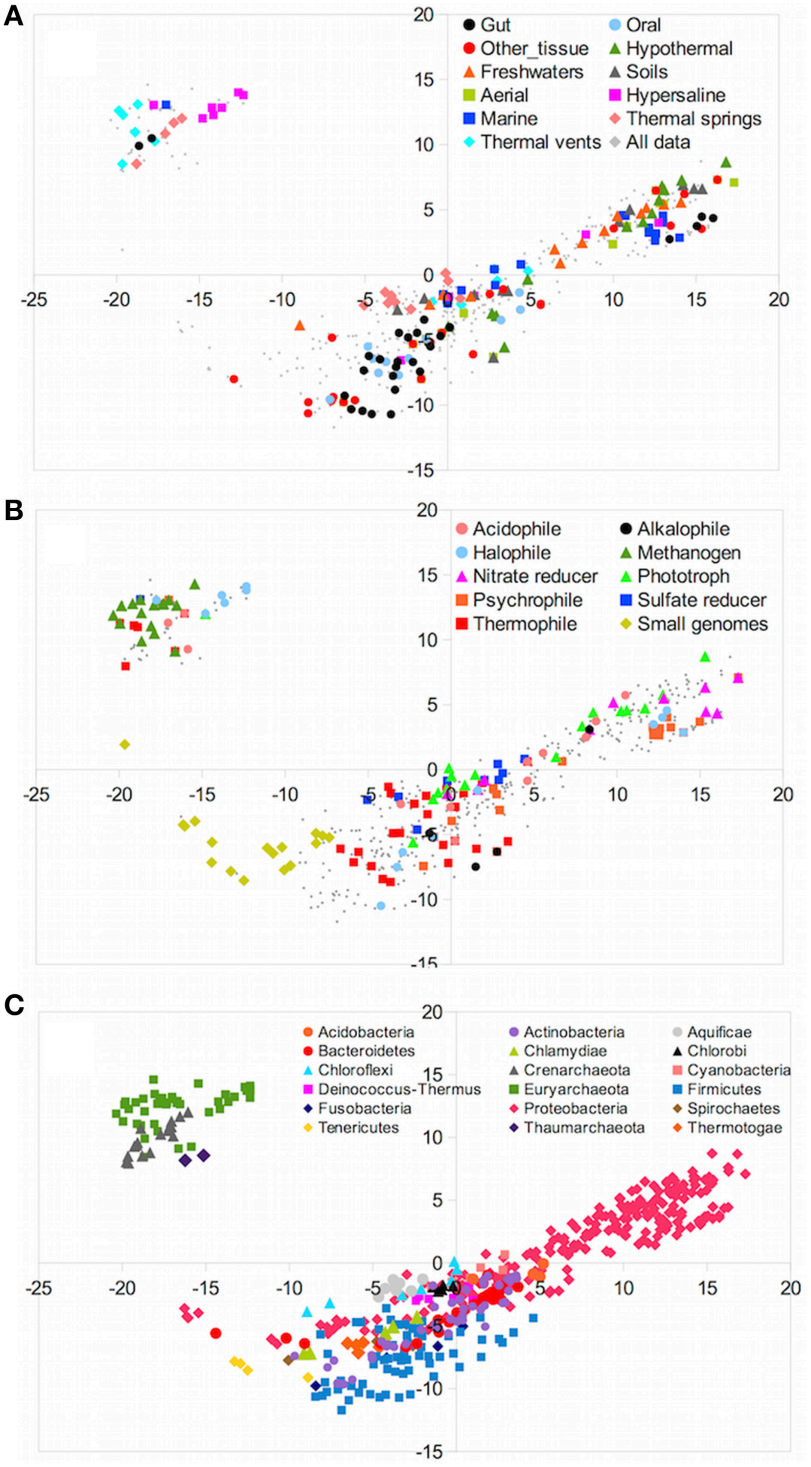
**MDS decomposition of the gene content matrix**. Each dot corresponds to a different genus, and these are colored according to different criteria in each plot. **(A)** colored by environmental preferences. **(B)** colored according to lifestyles. **(C)** colored according taxonomy.

First, we analyzed the similarities between taxa according to their gene content only. Figure 2 shows a multidimensional scaling (MDS) analysis of the gene content matrix. MDS attempts to reduce the n-dimensional genomic vectors, fitting them in two dimensions. Spatial proximity in the representation reflects similarity between gene content of the different genera. We color-coded the MDS decompositions using different criteria. Figure 2A colors the genera according to environmental preferences. It was apparent that genera preferring the same environments did not form a single cluster, indicating that inhabitants of the same environments could have different gene contents.

Lifestyles could have a more direct relationship to gene content than environmental preferences, as seen in Figure 2B. For instance, many thermophilic species mapped close to each other, indicating a high degree of genetic similarity. Nevertheless, some thermophiles were found in different parts of the diagram. The same trend could be seen for methanogenic or halophilic taxa and, especially, for taxa with small, reduced genomes. The latter have a rather different gene content because of the elimination of many genes, and therefore they clustered together and apart from the rest.

When plotting the data according to their taxonomy (Figure 2C), it was very clear that gene content was mostly driven by this factor. Genera belonging to the same phyla clustered relatively close to each other, although some large groups such as Proteobacteria showed high dispersion, indicating a wide range of possible metabolisms within them. The conspicuous separation into two main groups was easily explained by the segregation between bacterial and archaeal taxa. Thus, some characteristics like methanogenesis that apparently was very distinctive between taxa (Figure 2B) were better explained by the fact that methanogenic taxa belong to the archaeal domain. This was consistent with the conclusion that genetic content was more influenced by phylogenetic proximity than by environmental preferences or lifestyles.

Since the segregation between archaea and bacteria was the strongest factor, the MDS analysis was repeated removing archaeal taxa (Figure S4 in the Supplementary Material), in order to analyze in more detail the relationships within the bacteria. The results supported the observed trend, with phylogenetic relationships explaining most of the differences. The details, however, were quite suggestive. Most taxa were found in four large groups. Firmicutes and Actinobacteria, the two main phyla of Gram-positive bacteria, appeared close to each other. *Chloroflexi* and *Deinococcus*-*Thermus* phyla also appeared in this part of the diagram. The Proteobacteria formed another very large group, but the different classes were also separated from each other (Figure S5 in the Supplementary Material). A third less clear group was in between these two main groups, formed by a variety of phyla including Cyanobacteria, Bacteroidetes, Chlorobi, Aquificae, Acidobacteria, and some Proteobacteria. The only exceptions to the phylogenetic grouping were phylogenetically unrelated genera with reduced genomes, such as some small Tenericutes, Proteobacteria, Chlamydiae, and Bacteroidetes that were found together in the same part of the diagram. Altogether, this indicated that genetic potential was heavily dependent on phylogeny.

**Figure 3:**
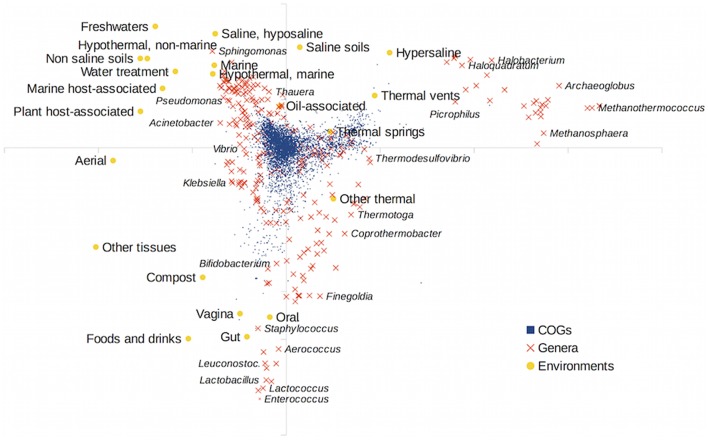
**Canonical Correspondence Analysis (CCA) of the gene content matrix, using environmental preferences as explanatory variables**. Orange crosses show the genera, blue squares the individual COGs in the matrix, and yellow circles represent the projections of the habitat preferences.

We also carried out a Canonical Correspondence Analysis of the gene content matrix using the environmental distribution of taxa as the external variable. The results are shown in Figure 3. Host-associated environments diverged very much from the other environments. The former environments impose a strong selection on the taxa that can live and thrive in them. As in the MDS analysis, archaeal taxa clustered separately (upper right corner of the figure). Since archaea are often associated with thermal and hypersaline habitats, these environments (thermal springs and vents, saline soils and hypersaline habitats) were well segregated in the analysis. A distinction between saline and non-saline environments was also apparent, and a gradient of increased salinity can be seen, from non-saline environments (like freshwaters) to hyposaline, saline and hypersaline habitats. This is in accordance with previous results taking into account just the environmental distribution of taxa (Lozupone and Knight, 2007; Tamames et al., 2010), in which temperature, salinity and association with host tissues were the most determinant environmental characteristics.

Some gene families (COGs plotted as blue squares in the figure) were closely associated with some environments. This was very apparent for host-associated environments and points to the existence of environmental-associated genes that could be linked to the successful adaptation to these environments. To determine the COGs that could be significantly enriched in some environments, we used the regression analysis implemented in the ShotgunFunctionalize R package (Kristiansson et al., 2009), relating the abundance of the particular COGS in taxa to the affinity of these taxa for the different environments (Figure S6 in the Supplementary Material). The most determinant COGs for several selected environments can be seen in Table S1 in the Supplementary Material. Genera in host-associated environments such as the gut were enriched mostly in metabolic genes related to utilization of carbohydrates, emphasizing the capability of these microbiotas to metabolize a wide range of complex organic compounds. Freshwater taxa, in turn, were enriched in cytochromes, especially in type *c* cytochromes. The other main characteristic of freshwater taxa was the abundance of branched-chain amino acid transport systems.

Because of the abundance of phototrophs in hypersaline environments such as salterns, we could find several genes related to this lifestyle, such as plastocyanins and bacteriorhodopsins. They were also enriched in archaeal genes. Also several proteins probably related to osmotic stress appeared in the list, like the universal stress protein UspA (Nyström and Neidhardt, 1994; Kim et al., 2013), or several ionic pumps.

Marine taxa were enriched in carbohydrate transport systems of the TRAP-type, for importing C4-dicarboxylates such as malate, fumarate, and succinate, perhaps to be used as carbon and energy sources (Rabus et al., 1999). Several of the genes found as overrepresented in saline/marine environments matched the most expressed genes in the proteome of the abundant marine bacterium *Pelagibacter*. For instance, TRAP transporters, sarcosine oxidase, spermidine/putrescine binding protein, and ABC sugar transporters (Sowell et al., 2009).

Thermal-associated genes are also biased because of the archaeal nature of many termophilic genera. Therefore, most marker genes corresponded to archaeal-specific genes, although there were also several antioxidant and repair proteins, perhaps related to an elevated risk of protein and DNA damage. Also, there were several genes related to methanogenesis, sulfate reduction, and hydrogenesis, metabolic processes that are very relevant in these thermal environments (Teske et al., 2003; Chou et al., 2008).

Altogether, the global content of genomes did not allow a separation according to their environmental preferences. At the genomic level, there was little correlation with environmental preferences. But such a correlation existed at the gene level, where individual genes were favored under particular conditions and were probable actors in the mechanisms of environmental adaptation.

### Part 2. quantification of the relationships between gene content, environmental preferences, and phylogeny

In this part, all genera are compared in pairs by measuring their similarities in gene content, environmental preferences, and phylogenetic distance. The first two measures are obtained by the calculation of a correlation coefficient between their vectors of gene content or environmental preferences. Phylogenetic distance is taken directly from the alignment of the respective 16S rRNA sequences of their constituent species. We also introduced a co-occurrence measure derived from the observation of co-occurring genera in environmental samples.

### Impact of environment and phylogeny on gene content

**Figure 4:**
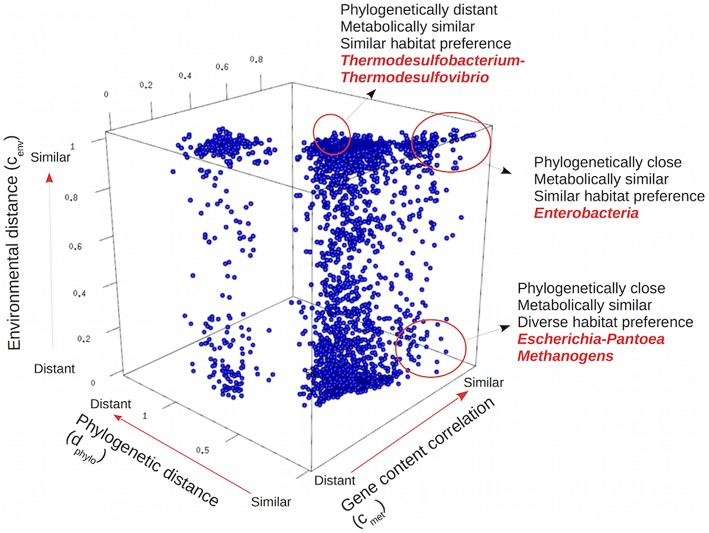
**Three-dimensional representation of genomic, environmental, and phylogenetic distances among all pairs of bacterial and archaeal genera**. Each point in the plot corresponds to a pair of genera, indicating their particular gene content, environmental and phylogenetic distances. Examples discussed in the text are highlighted.

We compared the pairs of genera by plotting their values of phylogenetic distance and environmental and gene content correlation, as shown in Figure 4. The two large clusters, corresponding to long or short phylogenetic distances, correspond to interdomain (between bacteria ad archaea) and intradomain (bacteria-bacteria or archaea-archaea) pairs, respectively. Phylogenetic closeness was usually associated with gene content similarity, but with a wide range of different environmental preferences. There were many instances of closely related organisms sharing similar environmental preferences, such as the many Enterobacteria taxa living preferentially in the digestive tract of animals, but closely related organisms could also diversify to live in different habitats. This was the case of *Pantoea*, a genus of the same Enterobacteria clade, and therefore closely related to the gut bacteria, but comprising pathogenic bacteria that can also be found on the surface of plants (Brady et al., 2008). Another example was methanogenic archaea, that despite being close phylogenetic relatives and metabolically similar, thrive in environments so different as anoxygenic sediments in the deep sea (genus *Methanobacterium)* and in the human gut (genus *Methanobrevibacter*; Liu and Whitman, 2008).

Examples of similar environmental preferences in very distant taxa could also be found. For instance, the genera *Thermodesulfobacterium* and *Thermodesulfovibrio* belong to different phyla (Thermodesulfobacteria and Nitrospirae respectively) but they share a similar life style in similar environments: they are both anaerobic, thermophilic, sulfate reducing bacteria (Muyzer and Stams, 2008).

**Figure 5:**
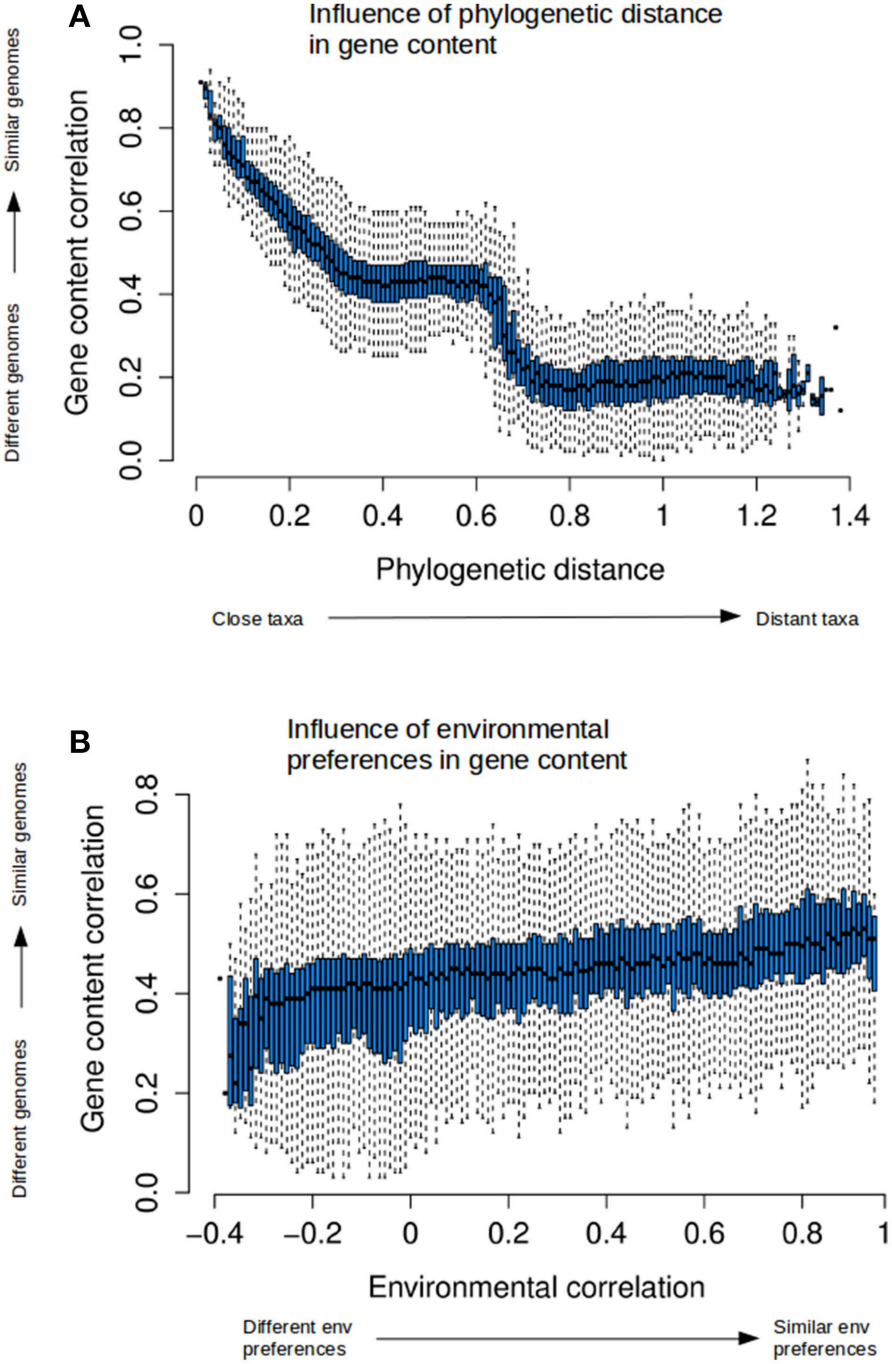
**Relationships between phylogenetic distance and gene content and environmental correlations**. Box-plots have been generated as explained in Figure S3, and show the quantification of the relationships between the three distances. Boxes are generated by discretizing the variable in the *x*-axis, and show the distribution of the measures in the *y*-axis corresponding to that discrete values in *x*. Therefore, the plots explain how the variable in *y* responds to the changes in *x*. Permuting the axes changes the discretization to the other variable. The boxes correspond to upper and lower quartiles of the data, and the marks within correspond to the median. Lines outside boxes (whiskers) show the variability outside the boxes, as an indication of the dispersion of the data. The plots shows how gene content similarity responds to: **(A)** phylogenetic distance, or **(B)** environmental correlation.

The relationships among these three measures were examined by looking separately at each side of the cube in Figure 4. The corresponding data are shown as box-plots in Figure 5 and Figures S9, S10 in the Supplementary Material (see Materials and methods and Figure S3 in the Supplementary Material for a detailed explanation of the creation of these box-plots). To aid in interpretation, Figure S7 in the Supplementary Material provides the correspondence between phylogenetic distance and taxonomic ranks.

Gene content was strongly related to phylogenetic distance (Figure 5A). The dispersion was low, indicating a very tight fit to the average trend. The sharp decline seen in the plots when phylogenetic distances reached values above 0.6 was again due to inter-domain comparisons (between bacteria and archaea, Figure S7 in the Supplementary Material), since members of different domains have the most distinct gene contents because of the presence of specific genes and even full pathways. The transposed plot (Figure S9B) indicated that taxa with similar genomic content were always phylogenetically close. We could not detect any events of metabolic convergence in which distant taxa had very similar gene contents.

The relationships between environmental correlation and phylogenetic distance showed that closely related taxa had a preference for living in similar environments, although this tendency decreased sharply (Figure S9C). The same data have been plotted in Figure S8 in the Supplementary Material adding the corresponding phylogenetic ranks to better reveal the correspondence between phylogenetic distances and these ranks, and the fact that the density of data points is not uniform along the phylogenetic distances. Most pairs fall at intermediate distances (between 0.2 and 0.5). Thus, the information for longer distances is dependent on less data and is not so robust.

In contrast to the relationship above, living in similar environments did not imply phylogenetically closeness (Figure S9D). Distant taxa had similar environmental preferences than closer ones, and there was no prevalence of closely related taxa in similar environments. Phylogenetic distance did not vary much along the full range of environmental correlations. This indicated that while closely related taxa tend to appear in the same environments (because, as remarked above, they have very similar metabolisms), they share these habitats with plenty of other taxa that are phylogenetically distant.

Figure 5B and Figure S9E illustrate the relationship between environmental preferences and gene content. There was a tendency for genera with similar genomes to be found in similar environments (Figure S9E), but the very high dispersion of the points indicated that taxa with very similar gene content could also be found in rather different environments. Examples of the latter are phototrophic organisms such as Cyanobacteria that can live in diverse habitats such as saline waters, freshwaters, soils, or even lichens. Also, taxa with unrelated genetic complement can have very similar environmental preferences. The other way around, taxa living in similar environments did not necessarily share the same gene content (Figure 5B). A wide range of gene contents are possible when living in similar environments, as indicated for the modest genetic correlation at higher environmental similarities, and for the wide dispersion all along the environmental correlation axis, implying the presence of both similar and dissimilar genetic contents. This reflects, for example, the associations between different guilds of organisms, such as thermophilic/halophilic bacteria and archaea in hydrothermal sources, or the synergistic associations of different taxa in the gut (Flint et al., 2008). There was a slight tendency to increase genetic similarity in response to environmental similarity, but it was much lower than that observed with phylogenetic distance (Figure 5A).

We also examined the trends relative to the co-occurrence of taxa in samples (Figure S10 in the Supplementary Material). Trends were similar to those found using environmental preferences. Notice that two taxa sharing environmental preferences do not necessarily co-occur (Figure S10E). They can even segregate, as in cases of competition. It is interesting to notice that phylogenetically close taxa tended to co-occur in samples (Figure S10A). Taxa with similar genomes are prone to co-occur (Figure S10C). This trend can correspond to a habitat-filtering model of interaction between taxa (Levy and Borenstein, 2013; Zelezniak et al., 2015). In contrast, co-occurrence did not drive a large increase in genomic correlation (Figure S10D). Most of the co-occurring taxa did not necessarily interact, but we could find examples of strongly co-occurring taxa with dissimilar metabolisms that are able to cooperate, such as the genuine interactions of the nitrifiers *Arthrobacter* with *Nitrobacter* denitrifiers, or some methanogenic archaea such as *Methanosaeta* with sulfate reducers like *Thermodesulfovibrio*, which even competing for hydrogen, can form a stable association under some conditions (Sekiguchi et al., 2008).

Altogether, the results indicate that gene content is highly related to phylogenetic closeness, and that the influence of environmental parameters is lower.

### Mantel tests

As a more robust test of the stronger connection of gene content with phylogeny than with environmental preferences, we performed Mantel tests of the fit between the matrices of genetic correlation and environmental correlations or phylogenetic distances, as well as co-occurrence strength. The Mantel test evaluates the correlation between two matrices A and B subtracting the correlation due to another matrix C and, therefore, it will determine whether genetic content fits better with phylogenetic distance than with environmental preferences.

**Figure 6:**
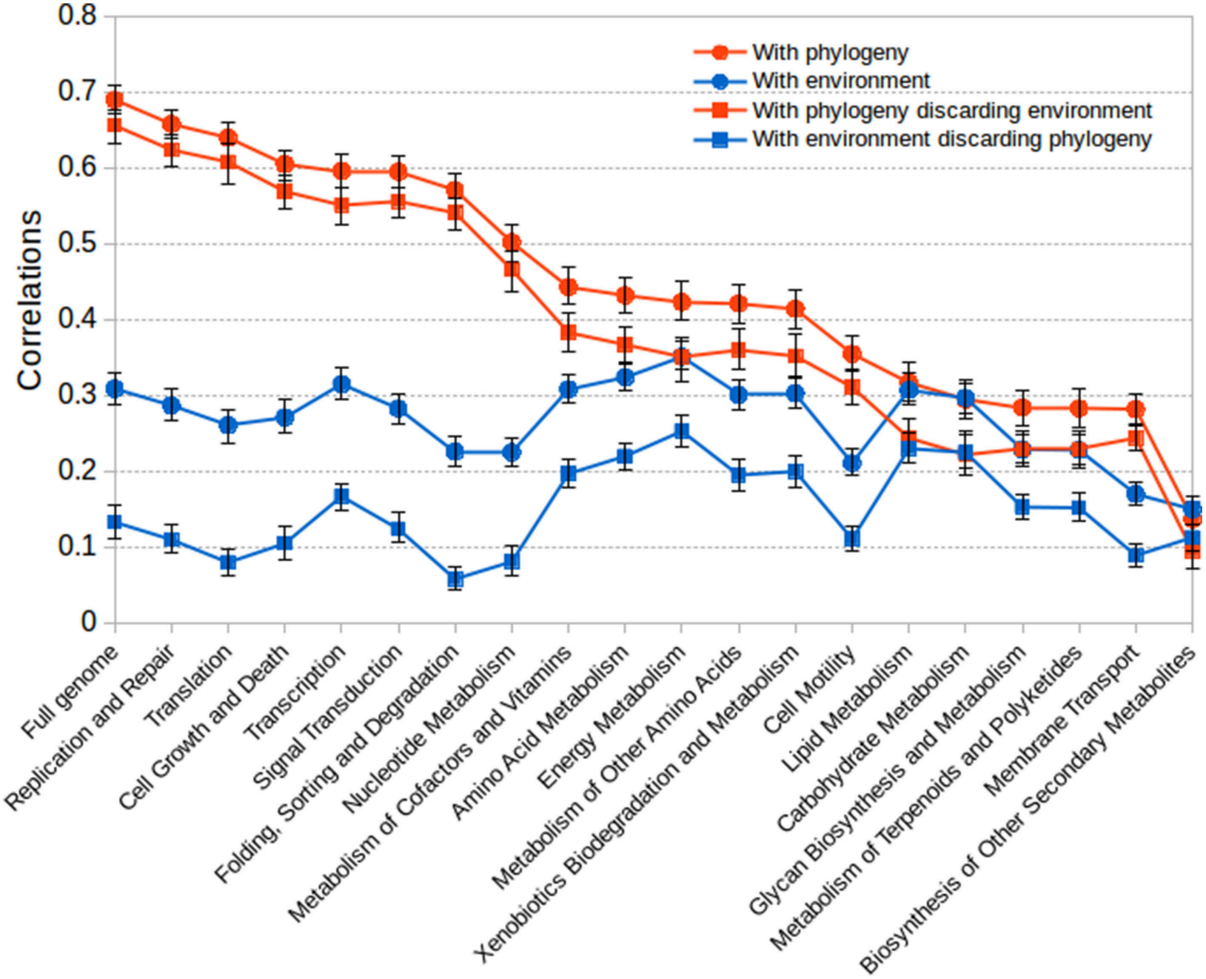
**Results for Mantel tests between matrices of gene content correlation and phylogenetic distance or environmental correlation**. The plot shows the fit between gene content correlation and phylogenetic distance, between gene content and environmental correlations, and the corresponding partial tests discounting either the influence of environmental correlation or the influence of phylogenetic distance. The bars show 95% confidence intervals.

Results are shown in Figure 6 for the full gene content and for sub-matrices calculated just using the genes corresponding to particular functional classes. In almost all cases the gene content correlated much better with the phylogeny than with environmental preferences. This again indicates that gene content is determined to a much greater extent by phylogenetic inheritance than by the adaptation to particular environments. Controlling for the influence of the environment with a partial Mantel test did not significantly affect the high correlation between phylogeny and gene content. Nevertheless, in some instances a substantial influence of the environment in gene content could be observed (right part of the graph in Figure 6). This was particularly true for some functional classes such as lipid metabolism, carbohydrate metabolism, glycan metabolism, energy metabolism and xenobiotic degradation, in which the influence of environment could be as high as (or even higher than) that of phylogeny.

To check that indeed core functions are much more phylogenetically determined, we included some non-metabolic classes like those related to management and processing of genetic information, such as replication, transcription and translation, or even cell cycle and cell growth. Our assumption was that these would be the classes less influenced by the environment and more related by phylogenetic inheritance, especially taking into account the difference between archaea and bacteria in some of these processes. Figure 6 shows that actually this was the case, with these processes showing a high correlation with the phylogeny and a very weak one (or null when discounting phylogenetic influence) with the environment (left part of the graph in Figure 6).

## Discussion

Previous studies attempting to describe the correlation between the phylogenetic relationships of microorganisms and their environmental preferences found that common habitat preferences were apparent below the taxonomic rank of class, and disappeared on that and ranks above (von Mering et al., 2007; Philippot et al., 2010). Our study supports and quantifies more precisely this trend, showing that while habitat preferences are shared strongly at lower taxonomic ranks (genus and family), there is still some amount of shared preferences for the class rank, that disappear at the phylum rank (Figure S8 in the Supplementary Material). This tendency is held for different levels of environmental classification and, more importantly, is supported also by co-occurrence data, a much more direct measure of environmental equivalence (Figure S10 in the Supplementary Material). These co-occurrences are greatly increased for lower taxonomic ranks, showing a propensity of close taxa to share environments and indicating that the process of speciation and divergence does not produce radical new environmental preferences.

It has been proposed that ecological coherence, that is, the uniformity of environmental preferences for a particular taxon, could be helpful to curate and support proposed taxonomies (Philippot et al., 2010). We show that this could be useful only for the lower taxonomic ranks such as genus and family. We also show that correlation in environmental preferences does not imply phylogenetic closeness, because several different lifestyles can be possible under the same conditions.

We have added a third perspective to this view of the relationships between phylogenetic relatedness and environmental preferences: the genomic content of the individual taxa. Phylogeny influences the genomic content by vertical inheritance: close relatives have close genomes. But this may be modified by the influence of the environment. Adaptation to a different environment is promoted by (and leads to) the acquisition of new genes for dealing with the novel conditions. In particular, genes such as specific transporters and regulators are often proposed as linked to adaptation (Boussau et al., 2004), and their acquisition can be accelerated via horizontal transfer (Ochman et al., 2000). An increased number of these genes can indeed indicate a higher potential for adaptability. A word of caution is advisable when studying the particular functions of these genes, since it is very difficult to predict the *in vivo* specificity of a given transporter, or the possible target of a regulator, just from the nucleotide sequences encoding them (Attisano and Wrana, 2002; Diallinas, 2014).

Therefore, genomes are composed by a mixture of core genes inherited mostly by vertical descent, and accessory genes related to adaptation that can be acquired and lost more easily. Our objective was to evaluate and quantify the contribution of these two factors, and to identify particular genes or processes that could be linked to environmental preferences. It has been shown that several taxa can be used as environmental markers because they are preferentially associated to some habitats (Lozupone and Knight, 2007; Tamames et al., 2010), but linking genes to environments has been more elusive up to now (Koonin and Wolf, 2008; Kastenmüller et al., 2009).

Our results show that genomic content is mostly related to phylogeny, but still there is some amount of variation due to the influence of the environment. Mantel tests show that this influence is located mainly in the accessory parts of the genomes. Functional classes such as membrane transport or secondary metabolism are the less determined by phylogeny but, perhaps surprisingly, they are not strongly influenced by environmental preference either. Instead, the classes that seem more linked to the environment are carbohydrate, lipid and glycan metabolisms, including their correspondent transporters. This points directly to resource availability as an important driving force in adaptation and shaping genomic content. For instance, host-associated bacteria, especially gut microorganisms, have a much bigger repertoire of genes for degrading carbohydrates and glycans, because these nutrients are more readily available in these environments (Koropatkin et al., 2012; El Kaoutari et al., 2013).

The task of deriving environmental marker genes, linking individual genes to different environmental preferences, was more successful for the environments that were more constrained, such as host-associated or thermal, than for others such as soils or marine, which are more diverse in the amount of niches in them. This is in accordance with our previous work showing that association to a host was the main selective characteristic (Tamames et al., 2010). Indeed, the microorganisms in these host-associated environments have an extensive toolkit for degradation of many different nutrients, as stated above. A handful of genes that were overrepresented in other environments could also be identified, even if explanation for the function is less straightforward. For instance, marine genomes are enriched in genes for small carbohydrates transport. We cannot determine the particular ecological role of these genes, but our observation is supported by the fact that these genes are also overrepresented in both marine metagenomes and proteomes (Figure S11 in the Supplementary Material). Also, it is possible that some of these genes are related to the gene content of other species (Fan et al., 2012), mediating direct or indirect interactions between them.

The possibility of identifying environmental marker genes allows to derive classifiers that can inform on the environmental preferences of sequenced species, in the way that was previously done for some habitats and phenotypic characteristics, like anaerobic or termophilic lifestyles (Kastenmüller et al., 2009). In some instances, it is possible to predict accurately the capability of a species to grow in particular environments just examining its genomic content. This could be helpful to engineer the habitat range of particular species.

The fact that most of the genomic content is determined vertically, by phylogenetic descent, explains why it is possible to derive functional profiles solely from the taxonomic content of microbial communities (Langille et al., 2013). This is a consequence of the close linkage between phylogeny and gene content, that allows to obtain these functional profiles from taxonomic assignments above the level of species. That is, having taxonomic information for genera or even families could be sufficient to obtain reasonably correct profiles. Nevertheless, one must be careful when following these approaches, since individual genes linked to environmental adaptations, probably the most interesting ones when studying the ecology of these microorganisms, will not be accurately predicted.

It can be argued that metagenomic data could be more appropriate for the purpose of detecting environmental favored genes than using individual genomes. Indeed, overrepresented genes in metagenomes have been used as environmental markers (Tringe et al., 2005; Dinsdale et al., 2008). The difference in our approach is that we wanted to focus on how environmental adaptation shapes individual genomes. That level of detail is not achievable with metagenomic data, where the abundance of genes is recorded irrespectively of their species of origin, and assignment of sequences to individual species is generally difficult and often impossible. Our study provides a different perspective that cannot be achieved with metagenomic data. Our approach is validated by the fact that environmental marker genes detected in our study are also well represented in the functional profiles obtained by metagenomic sequencing and analysis.

The research presented here was conducted in the absence of any commercial or financial relationships that could be construed as a potential conflict of interest.

## Author contributions

JT conceived and designed the study. JT and PS generated all data. JT and CP analyzed the results. JT, CP, and PIN raised the conclusions and drafted the manuscript.

## Funding

This work was supported by project CTM2013-48292-C3 (Ministerio de Economía y Competitividad, Spain).

### Conflict of interest statement

The authors declare that the research was conducted in the absence of any commercial or financial relationships that could be construed as a potential conflict of interest.
