# Supplementary material for: Quantifying the Relative Importance of Phylogeny and Environmental Preferences As Drivers of Gene Content in Prokaryotic Microorganisms
Source: Front Microbiol. 2016 Mar 31;7:433. doi: 10.3389/fmicb.2016.00433 (PMC4814473; doi:10.3389/fmicb.2016.00433)
Supplement: Supplementary file 1 [file Table1.DOCX]

| Environment | p-value | COG | Function | Functional class |
| --- | --- | --- | --- | --- |
| Gut | 6.60E-023 | COG0534 | Na+-driven multidrug efflux pump |  |
| Gut | 2.77E-022 | COG3250 | Beta-galactosidase/beta-glucuronidase | Carbohydrate transport and metabolism |
| Gut | 1.62E-021 | COG2207 | AraC-type DNA-binding domain-containing proteins | Transcription |
| Gut | 1.30E-020 | COG3525 | N-acetyl-beta-hexosaminidase | Carbohydrate transport and metabolism |
| Gut | 1.30E-020 | COG3537 | Putative alpha-1,2-mannosidase | Carbohydrate transport and metabolism |
| Gut | 9.17E-018 | COG0463 | Glycosyltransferases involved in cell wall biogenesis | Cell envelope biogenesis, outer membrane |
| Gut | 2.42E-017 | COG3119 | Arylsulfatase A and related enzymes | Inorganic ion transport and metabolism |
| Gut | 1.70E-015 | COG4974 | Site-specific recombinase XerD | DNA replication, recombination and repair |
| Gut | 2.35E-015 | COG4771 | Outer membrane receptor for ferrienterochelin and colicins | Inorganic ion transport and metabolism |
| Gut | 3.94E-014 | COG3712 | Fe2+-dicitrate sensor, membrane component | Signal transduction mechanisms\|Inorganic ion transport and metabolism |
| Gut | 2.44E-013 | COG4206 | Outer membrane cobalamin receptor protein | Coenzyme metabolism |
| Gut | 8.88E-013 | COG0577 | ABC-type antimicrobial peptide transport system, permease component |  |
| Gut | 1.20E-012 | COG3279 | Response regulator of the LytR/AlgR family | Signal transduction mechanisms\|Transcription |
| Gut | 3.76E-011 | COG3507 | Beta-xylosidase | Carbohydrate transport and metabolism |
| Gut | 9.80E-011 | COG1595 | DNA-directed RNA polymerase specialized sigma subunit, sigma24 homolog | Transcription |
| Gut | 3.81E-010 | COG3943 | Virulence protein | General function prediction only |
| Gut | 1.12E-009 | COG3579 | Aminopeptidase C | Amino acid transport and metabolism |
| Gut | 4.49E-009 | COG3669 | Alpha-L-fucosidase | Carbohydrate transport and metabolism |
| Gut | 7.45E-009 | COG3475 | LPS biosynthesis protein | Cell envelope biogenesis, outer membrane |
| Gut | 7.76E-009 | COG2244 | Membrane protein involved in the export of O-antigen and teichoic acid | General function prediction only |
| Gut | 1.67E-008 | COG5545 | Predicted P-loop ATPase and inactivated derivatives | General function prediction only |
| Gut | 6.47E-008 | COG3274 | Uncharacterized protein conserved in bacteria | Function unknown |
| Gut | 1.74E-007 | COG0738 | Fucose permease | Carbohydrate transport and metabolism |
| Gut | 1.94E-007 | COG1373 | Predicted ATPase (AAA+ superfamily) | General function prediction only |
| Gut | 3.98E-007 | COG3275 | Putative regulator of cell autolysis | Signal transduction mechanisms |
| Gut | 1.14E-006 | COG1472 | Beta-glucosidase-related glycosidases | Carbohydrate transport and metabolism |
| Gut | 1.86E-006 | COG4690 | Dipeptidase | Amino acid transport and metabolism |
| Gut | 3.33E-006 | COG0561 | Predicted hydrolases of the HAD superfamily | General function prediction only |
| Gut | 3.58E-006 | COG1649 | Uncharacterized protein conserved in bacteria | Function unknown |
| Gut | 1.11E-005 | COG0793 | Periplasmic protease | Cell envelope biogenesis, outer membrane |
| Gut | 1.47E-005 | COG2755 | Lysophospholipase L1 and related esterases | Amino acid transport and metabolism |
| Gut | 1.78E-005 | COG4299 | Uncharacterized conserved protein | Function unknown |
| Gut | 2.18E-005 | COG3345 | Alpha-galactosidase | Carbohydrate transport and metabolism |
| Gut | 2.29E-005 | COG1453 | Predicted oxidoreductases of the aldo/keto reductase family | General function prediction only |
| Gut | 4.07E-005 | COG3968 | Uncharacterized protein related to glutamine synthetase | General function prediction only |
| Gut | 4.82E-005 | COG3568 | Metal-dependent hydrolase | General function prediction only |
| Gut | 4.82E-005 | COG1368 | Phosphoglycerol transferase and related proteins, alkaline phosphatase | Cell envelope biogenesis, outer membrane |
| Gut | 5.08E-005 | COG2985 | Predicted permease | General function prediction only |
| Gut | 5.15E-005 | COG0110 | Acetyltransferase (isoleucine patch superfamily) | General function prediction only |
| Gut | 9.89E-005 | COG3757 | Lyzozyme M1 (1,4-beta-N-acetylmuramidase) | Cell envelope biogenesis, outer membrane |
| Freshwaters | 8.56E-010 | COG5001 | Predicted signal transduction protein | Signal transduction mechanisms |
| Freshwaters | 2.19E-006 | COG4654 | Cytochrome c551/c552 | Energy production and conversion |
| Freshwaters | 2.32E-004 | COG2863 | Cytochrome c553 | Energy production and conversion |
| Freshwaters | 5.94E-004 | COG0410 | ABC-type branched-chain amino acid transport systems, ATPase component | Amino acid transport and metabolism |
| Freshwaters | 8.01E-004 | COG2823 | Predicted periplasmic or secreted lipoprotein | General function prediction only |
| Freshwaters | 1.21E-003 | COG4566 | Response regulator | Signal transduction mechanisms |
| Freshwaters | 1.67E-003 | COG0559 | Branched-chain amino acid ABC-type transport system, permease components | Amino acid transport and metabolism |
| Freshwaters | 5.19E-003 | COG0625 | Glutathione S-transferase | Posttranslational modification, protein turnover, chaperones |
| Freshwaters | 5.75E-003 | COG0411 | ABC-type branched-chain amino acid transport systems, ATPase component | Amino acid transport and metabolism |
| Freshwaters | 6.08E-003 | COG1024 | Enoyl-CoA hydratase/carnithine racemase | Lipid metabolism |
| Freshwaters | 6.70E-003 | COG4177 | ABC-type branched-chain amino acid transport system, permease component | Amino acid transport and metabolism |
| Freshwaters | 9.24E-003 | COG3658 | Cytochrome b | Energy production and conversion |
| Freshwaters | 9.51E-003 | COG1538 | Outer membrane protein | Cell envelope biogenesis, outer membrane |
| Non-saline soils | 1.70E-013 | COG1680 | Beta-lactamase class C and other penicillin binding proteins |  |
| Non-saline soils | 4.00E-013 | COG1024 | Enoyl-CoA hydratase/carnithine racemase | Lipid metabolism |
| Non-saline soils | 4.00E-013 | COG2141 | Coenzyme F420-dependent N5,N10-methylene tetrahydromethanopterin reductase | Energy production and conversion |
| Non-saline soils | 6.89E-012 | COG1846 | Transcriptional regulators | Transcription |
| Non-saline soils | 1.49E-011 | COG1012 | NAD-dependent aldehyde dehydrogenases | Energy production and conversion |
| Non-saline soils | 1.72E-011 | COG2124 | Cytochrome P450 | Secondary metabolites biosynthesis, transport and catabolism |
| Non-saline soils | 1.77E-011 | COG2207 | AraC-type DNA-binding domain-containing proteins | Transcription |
| Non-saline soils | 1.16E-010 | COG0318 | Acyl-CoA synthetases (AMP-forming)/AMP-acid ligases II | Secondary metabolites biosynthesis, transport and catabolism\|Lipid metabolism |
| Non-saline soils | 7.49E-010 | COG0515 | Serine/threonine protein kinase | DNA replication, recombination and repair\|Transcription\|Signal transduction mechanisms\|General function prediction only |
| Non-saline soils | 7.97E-010 | COG4249 | Uncharacterized protein containing caspase domain | General function prediction only |
| Non-saline soils | 2.58E-009 | COG1529 | Aerobic-type carbon monoxide dehydrogenase, large subunit | Energy production and conversion |
| Non-saline soils | 3.78E-009 | COG2080 | Aerobic-type carbon monoxide dehydrogenase, small subunit | Energy production and conversion |
| Non-saline soils | 4.00E-009 | COG0154 | Asp-tRNAAsn/Glu-tRNAGln amidotransferase A subunit and related amidases | Translation, ribosomal structure and biogenesis |
| Non-saline soils | 2.55E-008 | COG3832 | Uncharacterized conserved protein | Function unknown |
| Non-saline soils | 2.59E-008 | COG2159 | Predicted metal-dependent hydrolase of the TIM-barrel fold | General function prediction only |
| Non-saline soils | 3.28E-008 | COG1506 | Dipeptidyl aminopeptidases/acylaminoacyl-peptidases | Amino acid transport and metabolism |
| Non-saline soils | 4.78E-008 | COG0625 | Glutathione S-transferase | Posttranslational modification, protein turnover, chaperones |
| Non-saline soils | 5.37E-008 | COG0654 | 2-polyprenyl-6-methoxyphenol hydroxylase and related oxidoreductases | Energy production and conversion\|Coenzyme metabolism |
| Non-saline soils | 8.76E-008 | COG0665 | Glycine/D-amino acid oxidases (deaminating) | Amino acid transport and metabolism |
| Non-saline soils | 3.10E-007 | COG3653 | N-acyl-D-aspartate/D-glutamate deacylase | Secondary metabolites biosynthesis, transport and catabolism |
| Non-saline soils | 7.37E-007 | COG2234 | Predicted aminopeptidases | General function prediction only |
| Non-saline soils | 7.97E-007 | COG1595 | DNA-directed RNA polymerase specialized sigma subunit, sigma24 homolog | Transcription |
| Non-saline soils | 9.06E-007 | COG3637 | Opacity protein and related surface antigens | Cell envelope biogenesis, outer membrane |
| Non-saline soils | 3.06E-006 | COG1802 | Transcriptional regulators | Transcription |
| Non-saline soils | 3.37E-006 | COG2936 | Predicted acyl esterases | General function prediction only |
| Non-saline soils | 3.76E-006 | COG2303 | Choline dehydrogenase and related flavoproteins | Amino acid transport and metabolism |
| Non-saline soils | 3.85E-006 | COG1172 | Ribose/xylose/arabinose/galactoside ABC-type transport systems, permease | Carbohydrate transport and metabolism |
| Non-saline soils | 4.03E-006 | COG0183 | Acetyl-CoA acetyltransferase | Lipid metabolism |
| Non-saline soils | 1.78E-005 | COG1228 | Imidazolonepropionase and related amidohydrolases | Secondary metabolites biosynthesis, transport and catabolism |
| Non-saline soils | 2.65E-005 | COG1879 | ABC-type sugar transport system, periplasmic component | Carbohydrate transport and metabolism |
| Non-saline soils | 3.19E-005 | COG0179 | 2-keto-4-pentenoate hydratase/2-oxohepta-3-ene-1,7-dioic acid hydratase | Secondary metabolites biosynthesis, transport and catabolism |
| Non-saline soils | 3.19E-005 | COG2514 | Predicted ring-cleavage extradiol dioxygenase | General function prediction only |
| Non-saline soils | 3.29E-005 | COG0715 | ABC-type nitrate/sulfonate/bicarbonate transport systems, periplasmic | Inorganic ion transport and metabolism |
| Non-saline soils | 3.29E-005 | COG2072 | Predicted flavoprotein involved in K+ transport | Inorganic ion transport and metabolism |
| Non-saline soils | 4.70E-005 | COG4221 | Short-chain alcohol dehydrogenase of unknown specificity | General function prediction only |
| Non-saline soils | 7.95E-005 | COG3485 | Protocatechuate 3,4-dioxygenase beta subunit | Secondary metabolites biosynthesis, transport and catabolism |
| Non-saline soils | 8.12E-005 | COG1129 | ABC-type sugar transport system, ATPase component | Carbohydrate transport and metabolism |
| Non-saline soils | 8.12E-005 | COG4993 | Glucose dehydrogenase | Carbohydrate transport and metabolism |
| Non-saline soils | 8.12E-005 | COG5616 | Predicted integral membrane protein | Function unknown |
| Hypersaline | 2.21E-042 | COG3413 | Predicted DNA binding protein | General function prediction only |
| Hypersaline | 3.38E-030 | COG1474 | Cdc6-related protein, AAA superfamily ATPase | Posttranslational modification, protein turnover, chaperones\|DNA replication, recombination and repair |
| Hypersaline | 4.27E-026 | COG0589 | Universal stress protein UspA and related nucleotide-binding proteins | Signal transduction mechanisms |
| Hypersaline | 1.71E-018 | COG1405 | Transcription initiation factor TFIIIB | Transcription |
| Hypersaline | 3.82E-015 | COG1378 | Predicted transcriptional regulators | Transcription |
| Hypersaline | 5.57E-014 | COG3794 | Plastocyanin | Energy production and conversion |
| Hypersaline | 1.41E-011 | COG4742 | Predicted transcriptional regulator | Transcription |
| Hypersaline | 1.41E-011 | COG3269 | Predicted RNA-binding protein, contains TRAM domain | General function prediction only |
| Hypersaline | 6.36E-008 | COG4250 | Predicted sensor protein/domain | Signal transduction mechanisms |
| Hypersaline | 1.66E-007 | COG2101 | TATA-box binding protein (TBP), component of TFIID and TFIIIB | Transcription |
| Hypersaline | 1.16E-006 | COG1599 | Single-stranded DNA-binding replication protein A (RPA) | DNA replication, recombination and repair |
| Hypersaline | 1.18E-006 | COG1522 | Transcriptional regulators | Transcription |
| Hypersaline | 1.33E-006 | COG5524 | Bacteriorhodopsin | General function prediction only |
| Hypersaline | 1.47E-006 | COG0569 | K+ transport systems, NAD-binding component | Inorganic ion transport and metabolism |
| Hypersaline | 3.86E-006 | COG1745 | Predicted metal-binding protein | General function prediction only |
| Hypersaline | 1.40E-005 | COG4711 | Predicted membrane protein | Function unknown |
| Hypersaline | 2.36E-005 | COG0630 | Type IV secretory pathway, involved in archaeal flagella biosynthesis | Cell motility and secretion |
| Hypersaline | 4.27E-005 | COG1943 | Transposase and inactivated derivatives | DNA replication, recombination and repair |
| Hypersaline | 4.33E-005 | COG2469 | Uncharacterized conserved protein | Function unknown |
| Hypersaline | 6.62E-005 | COG0704 | Phosphate uptake regulator | Inorganic ion transport and metabolism |
| Hypersaline | 7.00E-005 | COG3372 | Uncharacterized conserved protein | Function unknown |
| Hypersaline | 8.51E-005 | COG1757 | Na+/H+ antiporter | Energy production and conversion |
| Hypersaline | 1.77E-004 | COG1885 | Uncharacterized protein conserved in archaea | Function unknown |
| Hypersaline | 4.24E-004 | COG1967 | Predicted membrane protein | Function unknown |
| Hypersaline | 5.81E-004 | COG0003 | Oxyanion-translocating ATPase | Inorganic ion transport and metabolism |
| Hypersaline | 6.83E-004 | COG1824 | Permease, similar to cation transporters | Inorganic ion transport and metabolism |
| Hypersaline | 7.52E-004 | COG1695 | Predicted transcriptional regulators | Transcription |
| Hypersaline | 7.52E-004 | COG1715 | Restriction endonuclease |  |
| Hypersaline | 7.84E-004 | COG0638 | 20S proteasome, alpha and beta subunits | Posttranslational modification, protein turnover, chaperones |
| Hypersaline | 7.86E-004 | COG3390 | Uncharacterized protein conserved in archaea | Function unknown |
| Hypersaline | 8.29E-004 | COG0613 | Predicted metal-dependent phosphoesterases (PHP family) | General function prediction only |
| Hypersaline | 8.66E-004 | COG2309 | Leucyl aminopeptidase (aminopeptidase T) | Amino acid transport and metabolism |
| Hypersaline | 9.54E-004 | COG2226 | Methylase involved in ubiquinone/menaquinone biosynthesis | Coenzyme metabolism |
| Hypersaline | 9.54E-004 | COG1202 | Superfamily II helicase, archaea-specific | General function prediction only |
| Hypersaline | 9.60E-004 | COG1955 | Archaeal flagella assembly protein J | Cell motility and secretion |
| Marine | 3.22E-006 | COG3839 | ABC-type sugar transport systems, ATPase components | Carbohydrate transport and metabolism |
| Marine | 3.22E-006 | COG1593 | TRAP-type C4-dicarboxylate transport system, large permease component | Carbohydrate transport and metabolism |
| Marine | 7.54E-006 | COG0404 | Glycine cleavage system T protein (aminomethyltransferase) | Amino acid transport and metabolism |
| Marine | 7.74E-006 | COG1638 | TRAP-type C4-dicarboxylate transport system, periplasmic component | Carbohydrate transport and metabolism |
| Marine | 1.80E-005 | COG5598 | Trimethylamine:corrinoid methyltransferase | Coenzyme metabolism |
| Marine | 4.50E-005 | COG4665 | TRAP-type mannitol/chloroaromatic compound transport system, permease | Secondary metabolites biosynthesis, transport and catabolism |
| Marine | 1.01E-004 | COG3090 | TRAP-type C4-dicarboxylate transport system, small permease component | Carbohydrate transport and metabolism |
| Marine | 1.56E-004 | COG0625 | Glutathione S-transferase | Posttranslational modification, protein turnover, chaperones |
| Marine | 1.56E-004 | COG0687 | Spermidine/putrescine-binding periplasmic protein | Amino acid transport and metabolism |
| Marine | 2.60E-004 | COG5001 | Predicted signal transduction protein | Signal transduction mechanisms |
| Marine | 4.61E-004 | COG4664 | TRAP-type mannitol/chloroaromatic compound transport, large permease | Secondary metabolites biosynthesis, transport and catabolism |
| Marine | 5.36E-004 | COG1280 | Putative threonine efflux protein | Amino acid transport and metabolism |
| Marine | 1.63E-003 | COG1176 | ABC-type spermidine/putrescine transport system, permease component I | Amino acid transport and metabolism |
| Thermal springs | 2.28E-009 | COG4949 | Uncharacterized membrane-anchored protein conserved in bacteria | Function unknown |
| Thermal springs | 2.22E-008 | COG1669 | Predicted nucleotidyltransferases | General function prediction only |
| Thermal springs | 2.78E-005 | COG0425 | Predicted redox protein, regulator of disulfide bond formation | Posttranslational modification, protein turnover, chaperones |
| Thermal springs | 1.71E-004 | COG1337 | Uncharacterized protein predicted to be involved in DNA repair | DNA replication, recombination and repair |
| Thermal springs | 8.00E-004 | COG2044 | Predicted peroxiredoxins | General function prediction only |
| Thermal springs | 9.86E-004 | COG3258 | Cytochrome c | Energy production and conversion |
| Thermal springs | 1.15E-003 | COG0723 | Rieske Fe-S protein | Energy production and conversion |
| Thermal springs | 1.29E-003 | COG0433 | Predicted ATPase | General function prediction only |
| Thermal springs | 2.08E-003 | COG1013 | Pyruvate:ferredoxin oxidoreductase, beta subunit | Energy production and conversion |
| Thermal springs | 2.17E-003 | COG2380 | Uncharacterized protein conserved in bacteria | Function unknown |
| Thermal springs | 2.48E-003 | COG1366 | Anti-anti-sigma regulatory factor (antagonist of anti-sigma factor) | Signal transduction mechanisms |
| Thermal springs | 2.83E-003 | COG0247 | Fe-S oxidoreductase | Energy production and conversion |
| Thermal springs | 3.50E-003 | COG3255 | Putative sterol carrier protein | Lipid metabolism |
| Thermal springs | 5.23E-003 | COG0843 | Heme/copper-type cytochrome/quinol oxidases, subunit 1 | Energy production and conversion |
| Thermal springs | 5.55E-003 | COG0674 | Pyruvate:ferredoxin oxidoreductase, alpha subunit | Energy production and conversion |
| Thermal springs | 5.57E-003 | COG1353 | Predicted hydrolase of the HD superfamily (permuted catalytic motifs) | General function prediction only |
| Thermal springs | 6.47E-003 | COG0509 | Glycine cleavage system H protein (lipoate-binding) | Amino acid transport and metabolism |
| Thermal springs | 7.42E-003 | COG1144 | Pyruvate:ferredoxin oxidoreductase, delta subunit | Energy production and conversion |
| Thermal springs | 9.87E-003 | COG2905 | Predicted signal-transduction protein | Signal transduction mechanisms |
| Thermal springs | 9.94E-003 | COG2368 | Aromatic ring hydroxylase | Secondary metabolites biosynthesis, transport and catabolism |
| Thermal vents | 3.92E-008 | COG1672 | Predicted ATPase (AAA+ superfamily) | General function prediction only |
| Thermal vents | 8.78E-007 | COG1331 | Highly conserved protein containing a thioredoxin domain | Posttranslational modification, protein turnover, chaperones |
| Thermal vents | 3.31E-006 | COG1848 | Predicted nucleic acid-binding protein, contains PIN domain | General function prediction only |
| Thermal vents | 1.26E-005 | COG2863 | Cytochrome c553 | Energy production and conversion |
| Thermal vents | 1.43E-005 | COG2202 | FOG: PAS/PAC domain | Signal transduction mechanisms |
| Thermal vents | 1.50E-003 | COG0731 | Fe-S oxidoreductases | Energy production and conversion |
| Thermal vents | 1.71E-003 | COG2995 | Uncharacterized paraquat-inducible protein A | Function unknown |
| Thermal vents | 1.88E-003 | COG1143 | Formate hydrogenlyase subunit 6/NADH:ubiquinone oxidoreductase | Energy production and conversion |
| Thermal vents | 4.10E-003 | COG1014 | Pyruvate:ferredoxin oxidoreductase, gamma subunit | Energy production and conversion |
| Thermal vents | 4.37E-003 | COG4314 | Predicted lipoprotein involved in nitrous oxide reduction | Energy production and conversion |
| Thermal vents | 5.70E-003 | COG0651 | Formate hydrogenlyase subunit 3/Multisubunit Na+/H+ antiporter | Inorganic ion transport and metabolism\|Energy production and conversion |
| Thermal vents | 5.70E-003 | COG2989 | Uncharacterized protein conserved in bacteria | Function unknown |
| Thermal vents | 6.52E-003 | COG0569 | K+ transport systems, NAD-binding component | Inorganic ion transport and metabolism |
| Thermal vents | 8.83E-003 | COG5001 | Predicted signal transduction protein | Signal transduction mechanisms |
| Thermal vents | 8.84E-003 | COG1013 | Pyruvate:ferredoxin oxidoreductase, beta subunit | Energy production and conversion |
