# Supplementary material for: Quantifying the Relative Importance of Phylogeny and Environmental Preferences As Drivers of Gene Content in Prokaryotic Microorganisms
Source: Front Microbiol. 2016 Mar 31;7:433. doi: 10.3389/fmicb.2016.00433 (PMC4814473; doi:10.3389/fmicb.2016.00433)
Supplement: Supplementary file 2 [file DataSheet1.DOCX]

Supplementary material

**Relationships between gene content, environmental preferences and phylogenetic divergence in prokaryotic microorganisms**

**Javier Tamames, Pablo D. Sánchez, Pablo I. Nikel, Carlos Pedrós-Alió**

Table S1: COGs overrepresented in different environments, according to the regression analysis implemented in ShotgunFunctionalizeR (Kristiansson *et al.*, 2009). The columns show the environment, COG id, COG function, corresponding functional classes, and the p-value of the association between the environment and the COG (adjusted for multiple testing)

Figure S1: Full description of the procedure used for generating the data. The first page shows a summary of the data types and their production. The rest of the pages describe in detail each of the steps. A: Production of gene content matrix. B: Production of the environmental preference matrix. C: Retrieval of phylogenetic distances. D: Derivation of gene content, environmental and co-occurrence distances. E: Grouping of all distances to produce the final combined distance matrix.

Figure S2: Number of annotated COGs for each of the complete genomes used in this study, in relation to genomic size. We observe a well-defined, non-linear trend, in which bigger genomes have proportionally fewer annotations, due to the abundance of hypothetical or unidentified genes in them. Points that fell out of this trend (shown in red) indicated genomes with less annotations and were removed from the analysis.

Figure S3: Creation of box-plots. The figures show the relationship between environmental and metabolic correlations for pairs of taxa. For each pair of species, we obtain a value for each of the two correlations that define a single point in the upper plots. Notice that data in the plots are identical, and the variation is just in the choice of x- or y-axis for each of the correlations. Since many points overlap, the data are shown as a density plot where darker colors correspond to higher number of points. For instance, we can see that there are many instances of zero value for environmental correlation and around 0.4 for gene content correlation. From these data, discretization of the x-axis in boxes originates the box-plots below. Plots are different even if they have been done using the same data: the one in the left informs on the variation of the gene content distance between taxa when environmental distance increases (when taxa tend to live in similar environments), while the one in the right shows how environmental distance vary in response to gene content distance (when taxa tend to have more similar genomes).

Figure S4: NMDS decomposition of gene content matrix when removing archaeal genera. Top: colored by environmental preferences. Center: colored according lifestyle. Bottom: colored according taxonomic assignment.

Figure S5: NMDS decomposition of gene content matrix when removing archaeal genera, discriminating within Proteobacteria.

Figure S6: Regression analysis of the abundance of COG3250 (Beta-galactosidase) in the gut environment. Each point of the plot indicates one genus. X-axis shows the affinity of these genera for the gut, and Y-axis indicated the relative abundance of COG3250 in each particular genus. Numbers above the points show the absolute abundance of COG3250 in each genus.

Figure S7: Corresponding values of phylogenetic distances for intra-rank comparisons. Phylogenetic distances for taxa belonging to same ranks (Domain, superkingdom, phylum, class, order, family and genus) are plotted as a density map (upper), in which brighter colors indicate higher density of points, and as a box-plot (lower), showing the median value of the distance for each rank.

Figure S8: Boxplot of the response of environmental distance to variation in phylogenetic distance (same plot than figure 3C), The width of the boxes indicates the amount of data in each box of phylogenetic distance, showing that most data are distributed between 0.2 and 0.5 in this axis. Corresponding phylogenetic ranks are depicted on the lower part of the plot. Green and red horizontal lines indicate the median and the average values of environmental distances, respectively.

Figure. S9**:** Relationships between phylogenetic distance and gene content and  environmental correlations. Box-plots have been generated as explained in Figure S3 and Figure 5. (A) This plot shows how gene content correlation responds to the increase in the phylogenetic distance. (B) Phylogenetic distance in response to gene content correlation. (C) Environmental correlation in response to phylogenetic distance. (D) Phylogenetic distance in response to environmental correlation. (E) Environmental  correlation in response to gene content correlation. (F) Gene content correlation in  response to environmental correlation.

Figure S10: Boxplots of co-occurrence data, as in Figure 3. A: This plot shows how co-occurrence strength responds to the variation in the phylogenetic distance. B: Phylogenetic distance in response to co-occurrence strength. C: Co-occurrence strength in response to gene content distance. D: Gene content distance in response to co-occurrence strength. E: Co-occurrence strength in response to environmental distance. F: Environmental distance in response to co-occurrence strength.

Figure S11: Comparison of the abundance of two particular COGs in gut (Blue bars) and marine (Orange bars) metagenomes. Upper: COG0404, Glycine cleavage system T protein (aminomethyltransferase). This function in overrepresented in marine metagenomes. Lower, COG3250, Beta-galactosidase. Intestinal metagenomes are enriched in this COG. Significance tests and plots were obtained using STAMP (http://kiwi.cs.dal.ca/Software/STAMP)
