# Supplementary material for: Quantifying the Relative Importance of Phylogeny and Environmental Preferences As Drivers of Gene Content in Prokaryotic Microorganisms
Source: Front Microbiol. 2016 Mar 31;7:433. doi: 10.3389/fmicb.2016.00433 (PMC4814473; doi:10.3389/fmicb.2016.00433)
Supplement: Supplementary file 3 [file Image1.PDF]

NCBI's complete genomes

COG assignment

META	COG0001	COG0002	COG0003
Acetobacter	1	0.3	1.5
Achromobacter	0.6	1.1	1
Bacillus	0	0.4	2

Gene Content Matrix

Spearman's correlation

Acetobacter	1	0.3	...
Achromobacter	0.6	1.1	...

Corr=0.41

GreenGenes 16S rRNA aligned sequences

>*Acetobacter aceti*
T-TGAA-C-GC—TGG-C--G-GC-A-TG--C----T
>*Achromobacter xylosoxidans*
-GGTACT-------------------AA--C--CT--T--AG-C
>*Bacillus anthracis*
AGAGTTTTGA--T-CC-T-G-GCTC-AG-AA-TG

RaxML distance calculation

PHYLO	*Acetobacter*	*Achromobacter*	*Bacillus*
Acetobacter	0	0.034	0.215
Achromobacter	0.034	0	0.493
Bacillus	0.215	0.493	0

c_{gen} d_{phyl}

Genus1	Genus 2	c_{gen}	d_{phyl}	c_{env}	s_{cooc}
Acetobacter	***Achromobacter***	**0.41**	**0.034**	**0.25**	**0**
Bacillus	***Geobacillus***	**0.74**	**0.12**	**0.32**	**12.87**
Haloquadratum	***Halorubrum***	**0.63**	**0.20**	**0.96**	**23.55**

COMBINED TABLE

Acetobacter	3.45	-1.2	...
Achromobacter	0.01	0.85	...

Corr=0.25

c_{env} s_{cooc}

Spearman's correlation

ENV	Forest	Gut	Ocean
Acetobacter	3.45	-1.2	0.03
Achromobacter	0.01	0.85	-0.03
Bacillus	-3.1	8.34	-1.82

Environmental Preference Matrix

Fisher's one-tailed test

	Forest	Gut	Ocean
Acetobacter	254	0	23
Achromobacter	9	43	1
Bacillus	0	645	0

Fisher's one-tailed test

COOC	*Acetobacter*	*Achromobacter*	*Bacillus*
Acetobacter	-	12	0
Achromobacter	12	-	5
Bacillus	0	5	-

	Sample1	Sample2	Sample3
Acetobacter	254	0	23
Achromobacter	9	43	1
Bacillus	0	645	0

GenBank environmental samples

SUMMARY

A. GENE CONTENT

NCBI's complete genomes

COG assignment

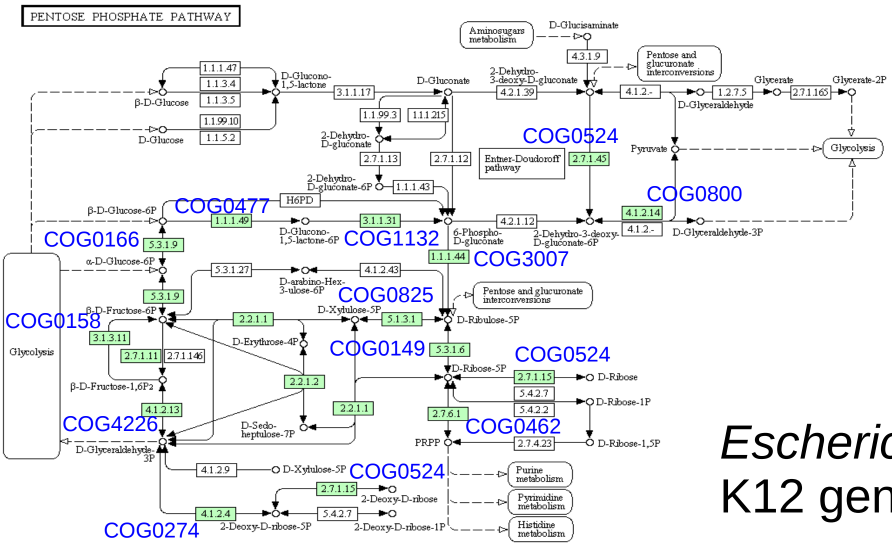

Escherichia coli
K12 genome

	COG0001	COG0002	COG0003
Genome 1 (Genus 1)	1	0	2
Genome 2 (Genus 1)	1	1	1
Genome 3 (Genus 2)	0	1	3

Taxonomic classification of genomes

COGs in genomes

Averaging by number of genomes in each genera

	COG0001	COG0002	COG0003
Genus 1	1	0.5	1.5
Genus 2	0.6	1.1	1
Genus 3	0	0.4	2

COGs in genera

B. ENVIRONMENTAL ASSOCIATIONS

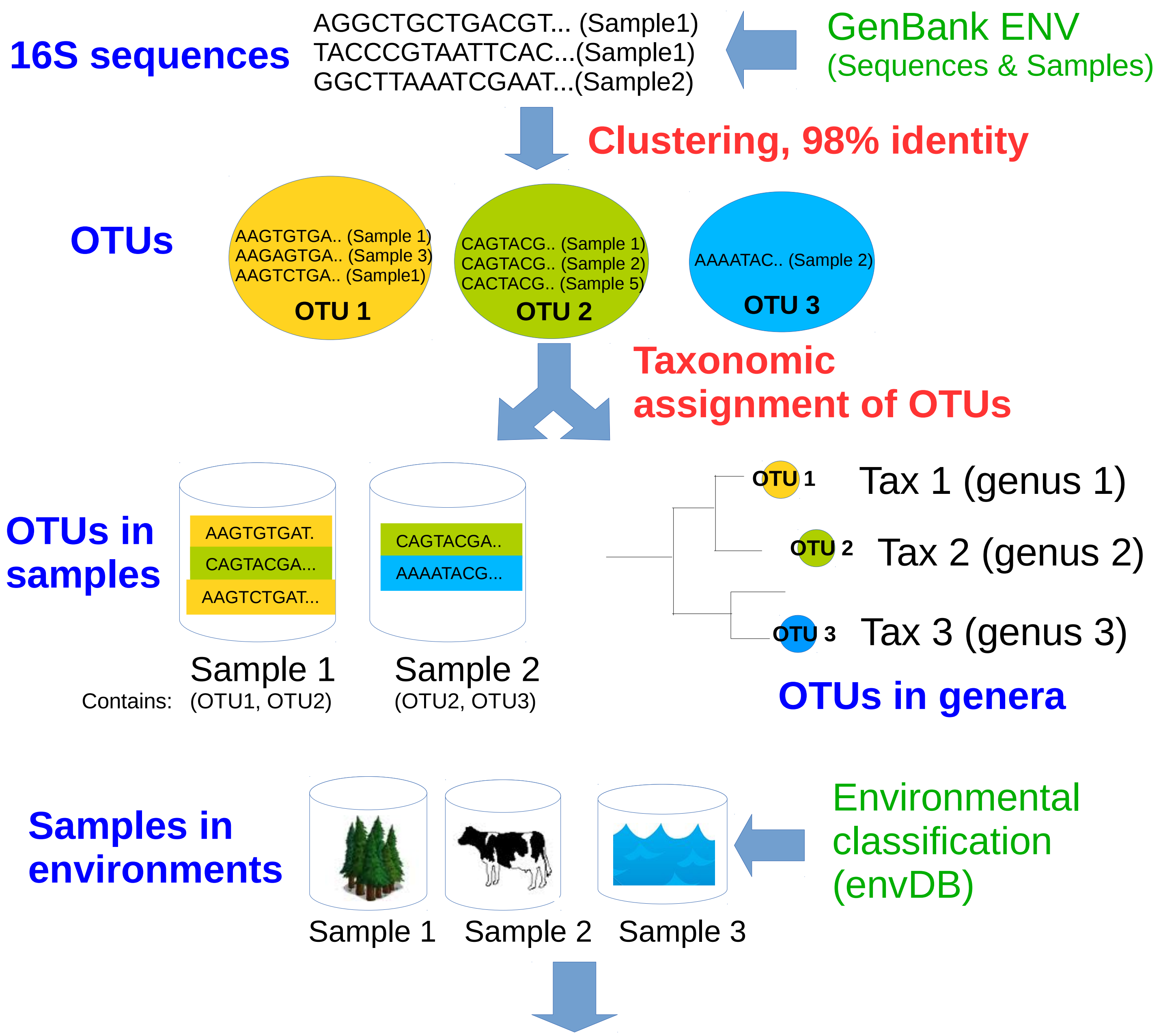

	Forest	Gut	Ocean
Genus 1	254	0	23
Genus 2	9	43	1
Genus 3	0	645	0

Taxa in environments

	Sample 1	Sample 2	Sample 3
Genus 1	18	0	2
Genus 2	3	5	0
Genus 3	0	21	0

Taxa in samples

Fisher's two-tailed test

	Forest	Gut	Ocean
Genus 1	3.45	-1.2	0.03
Genus 2	0.01	0.85	-0.03
Genus 3	-3.1	8.34	-1.82

Associations taxa-environments

	Genus 1	Genus 2	Genus 3
Genus 1	-	12	0
Genus 2	12	-	5
Genus 3	0	5	-

Co-ocurrences matrix

C. PHYLOGENETIC DISTANCES

GreenGenes 16S rRNA aligned sequences

>44919 AF310437.1 Neisseria meningitidis str. M1976
T-TGAA-C-GC—TGG-C--G-GC-A-TG--C----T-T--TACACA-T-GC-A-AGT-CGG-A-CG----------G-CAG-CA-C

>87220 AY289925.1 Acinetobacter calcoaceticus ADP1
-GGTACT-------------------AA--C--CT--T--AG-C-GG-C-GG-A--C-------------GGG-TGAGT-A--AT-AC-T-T-A-GG---

>22537 AF395031.1 Sphingomonas sp. str. M3C203B-B
AGAGTTTTGA--T-CC-T-G-GCTC-AG-AA-TGAA-C-GC--TGG-C--G-GC-A-TG--C----C-T--AACACA-T-GC-A-A

RaxML distance calculation

	Species 1 (Genus 1)	Species 2 (Genus 1)	Species 3 (Genus 2)
Species 1 (Genus 1)	-	0.012	0.232
Species 2 (Genus 1)	0.012	-	0.439
Species 3 (Genus 2)	0.232	0.439	-

Averaging distances by genus

d_{phylo}	Genus 1	Genus 2	Genus 3
Genus1	0.002	0.034	0.215
Genus 2	0.034	0	0.493
Genus 3	0.215	0.493	0

Phylogenetic distance matrix

D. DISTANCE MATRICES

	Forest	Gut	Ocean
Genus 1	3.45	-1.2	0.03
Genus 2	0.01	0.85	-0.03
Genus 3	-3.1	8.34	-1.82

Associations taxa-environments
(Env preferences matrix)

	COG0001	COG0002	COG0003
Genus 1	1	0.3	1.5
Genus 2	0.6	1.1	1
Genus 3	0	0.4	2

COGs in genera
(Gene content matrix)

Spearman's correlation between genera vectors

Genus 1	1	0.3	1.5	...
Genus 2	0.6	1.1	1	...

Corr=0.857

c_{env}	Genus 1	Genus 2	Genus 3
Genus1	-	0.745	0.423
Genus 2	0.745	-	0.521
Genus 3	0.423	0.745	-

Environmental correaltion matrix

c_{gen}	Genus 1	Genus 2	Genus 3
Genus1	-	0.857	0.503
Genus 2	0.857	-	0.551
Genus 3	0.503	0.551	-

Genetic correlation matrix

	Genus 1	Genus 2	Genus 3
Genus 1	-	12	0
Genus 2	12	-	5
Genus 3	0	5	-

Co-ocurrences matrix

Fisher's one-tailed test

s_{cooc}	Genus 1	Genus 2	Genus 3
Genus 1	-	2.53	0
Genus 2	2.53	-	0.72
Genus 3	0	0.72	-

Co-ocurrence strength matrix

---

d_{phylo}	Genus 1	Genus 2	Genus 3
Genus1	0.002	0.034	0.215
Genus 2	0.034	0	0.493
Genus 3	0.215	0.493	0

Phylogenetic distance matrix (from section C)

E. COMBINED MATRIX

Genus1	Genus 1	c_{gen}	d_{phyl}	c_{env}	s_{cooc}
Acetobacter	*Achromobacter*	0.41	0.31	0.25	0
Bacillus	*Geobacillus*	0.74	0.12	0.32	12.87
Haloquadratum	*Halorubrum*	0.63	0.20	0.96	23.55
Enterobacter	*Haloquadratum*	0.10	1.11	-0.15	0
Escherichia	*Salmonella*	0.88	0.04	0.97	20.58
Desulfovibrio	*Methanosarcina*	0.14	0.92	0.71	2.46

Combined distance matrix
