# Supplementary figures and images for: Quantifying the Relative Importance of Phylogeny and Environmental Preferences As Drivers of Gene Content in Prokaryotic Microorganisms
Source: Front Microbiol. 2016 Mar 31;7:433. doi: 10.3389/fmicb.2016.00433 (PMC4814473; doi:10.3389/fmicb.2016.00433)

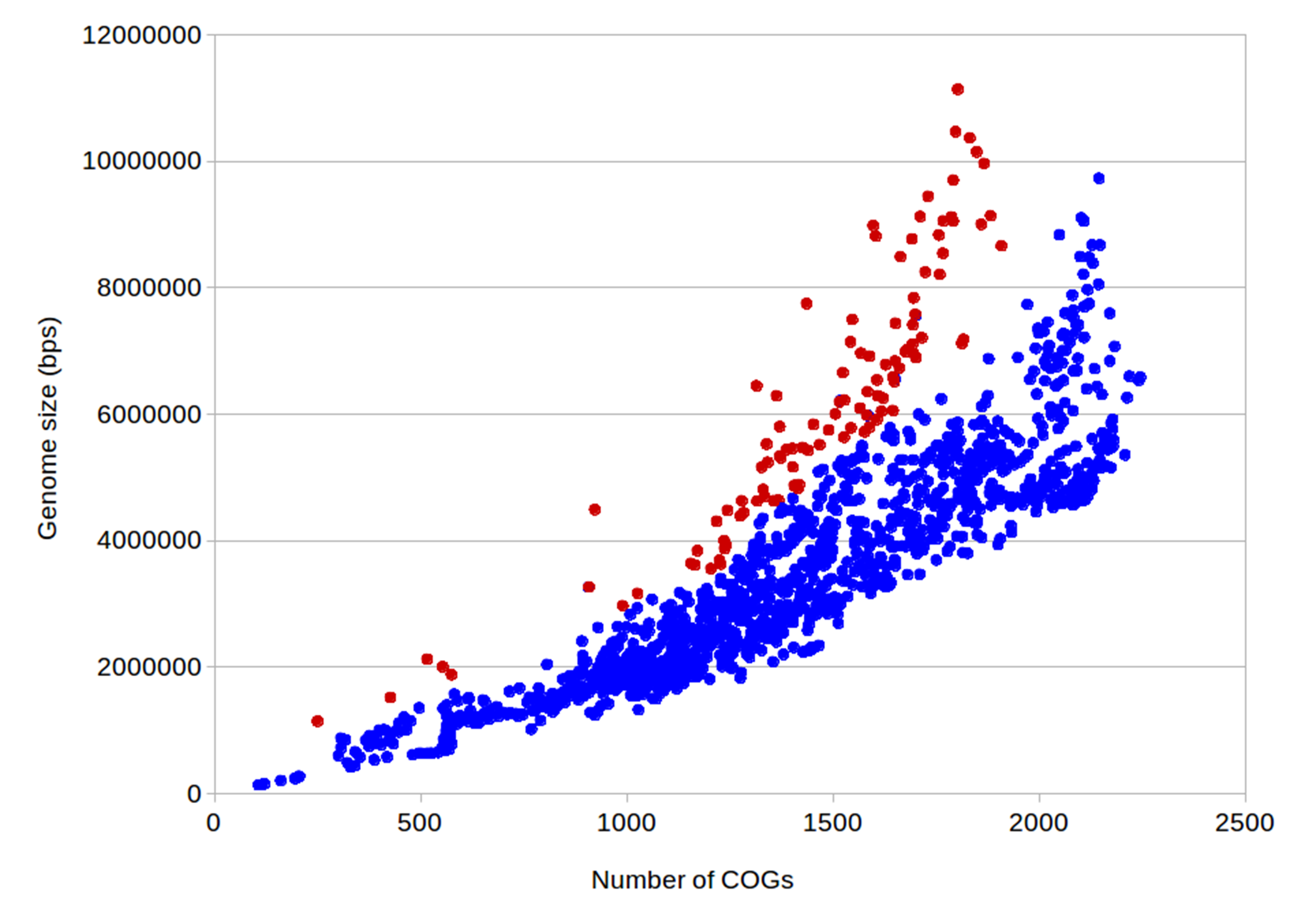

Supplement: Supplementary file 4 [file Image2.TIF]

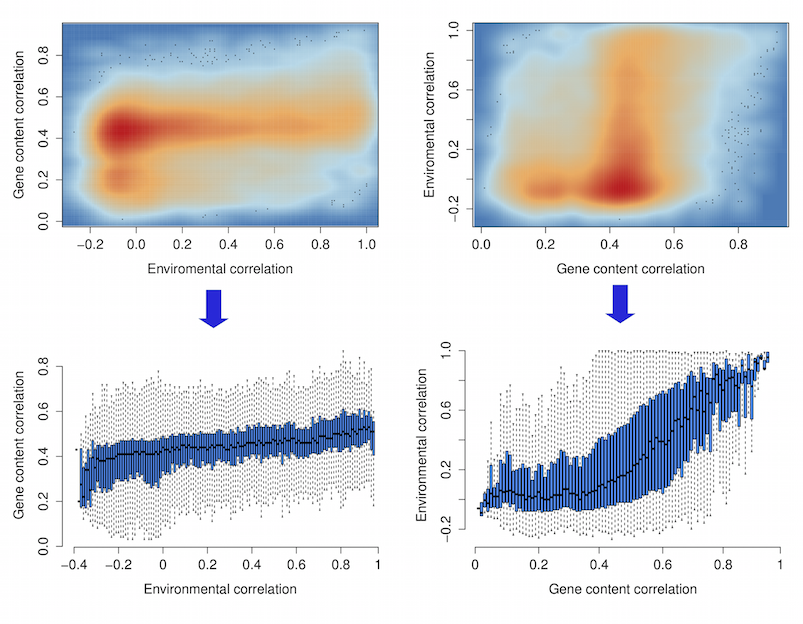

Supplement: Supplementary file 5 [file Image3.TIFF]

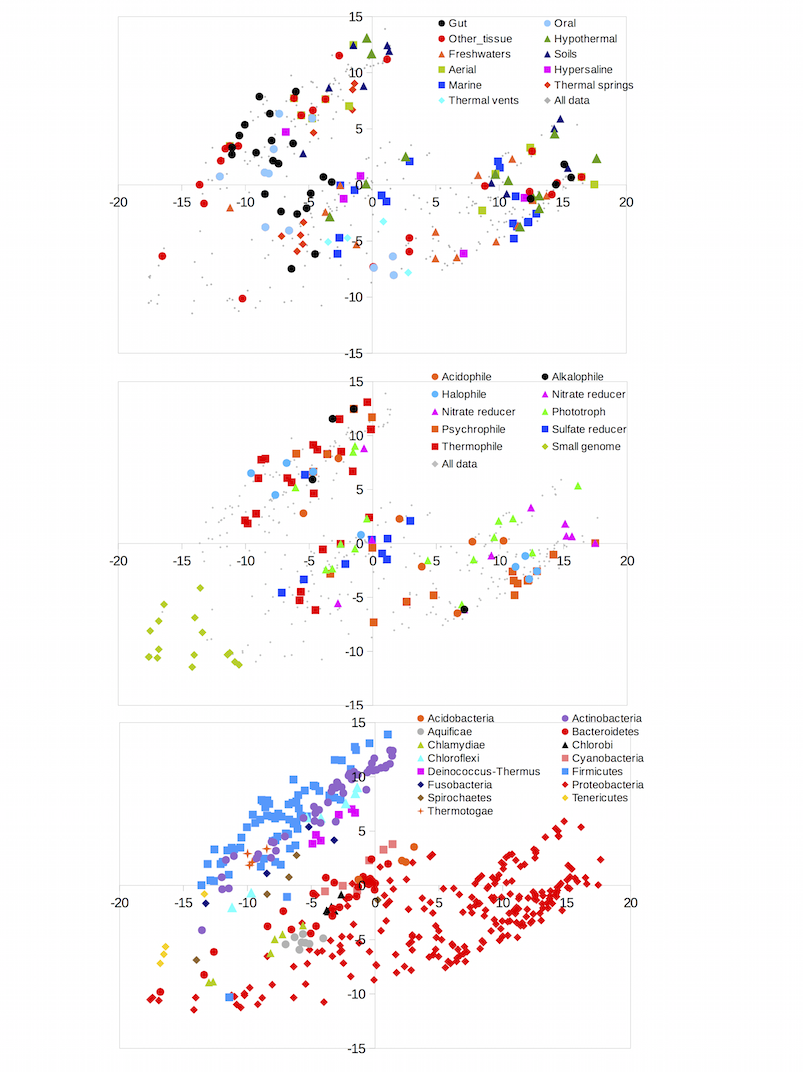

Supplement: Supplementary file 6 [file Image4.TIFF]

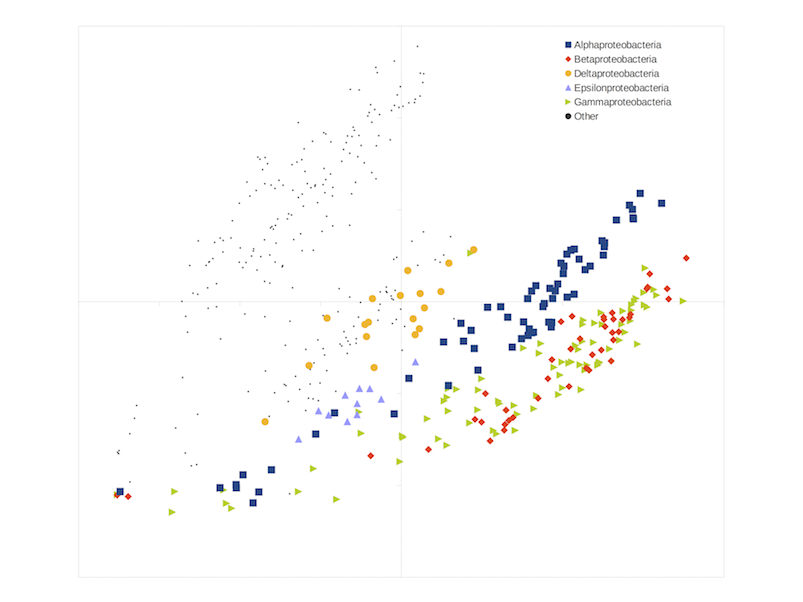

Supplement: Supplementary file 7 [file Image5.TIFF]

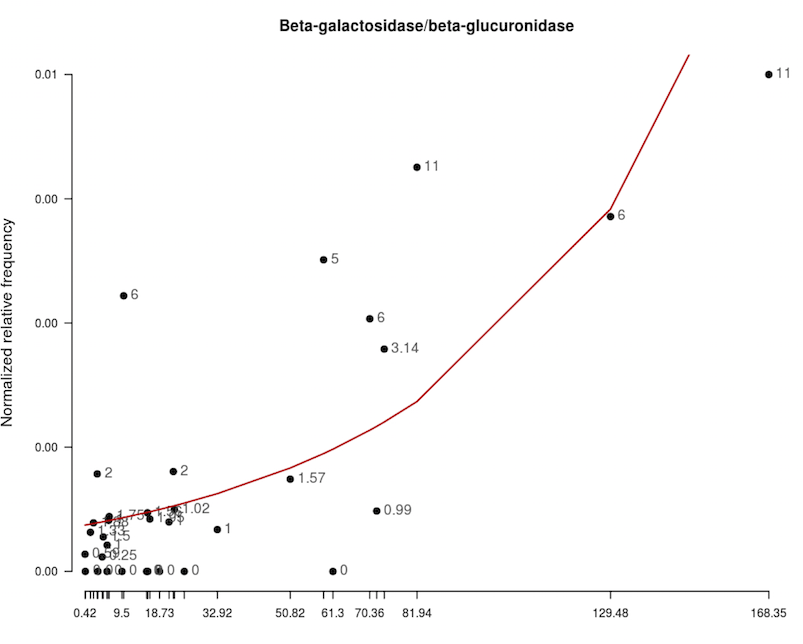

Supplement: Supplementary file 8 [file Image6.TIFF]

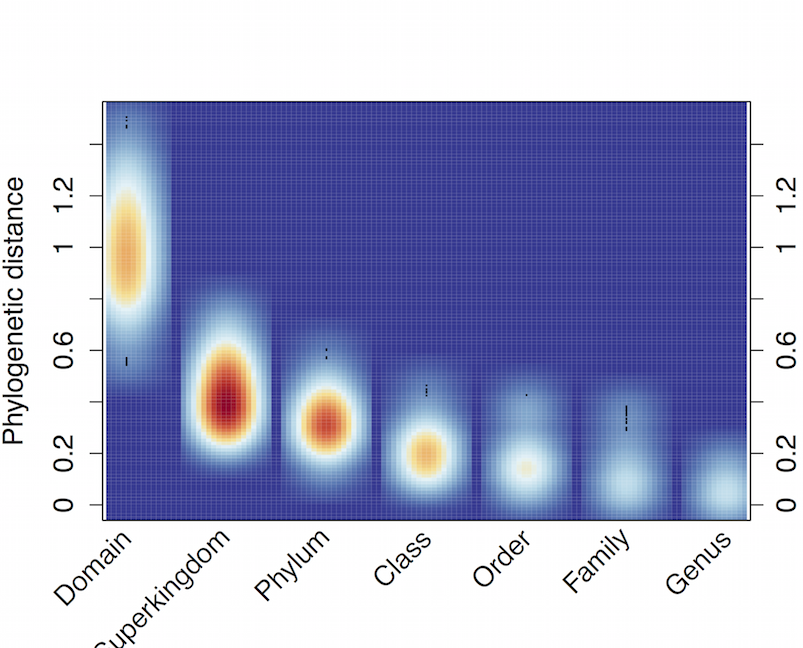

Supplement: Supplementary file 9 [file Image7.TIFF]

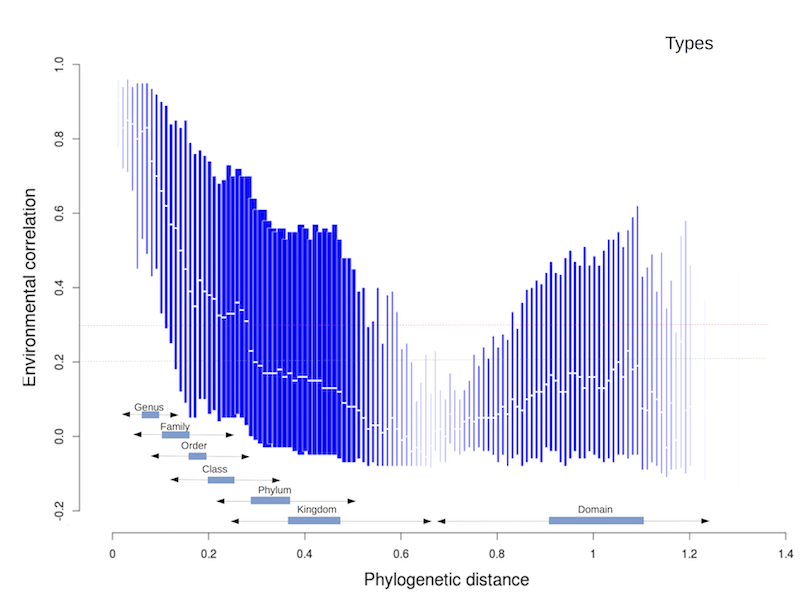

Supplement: Supplementary file 10 [file Image8.JPEG]

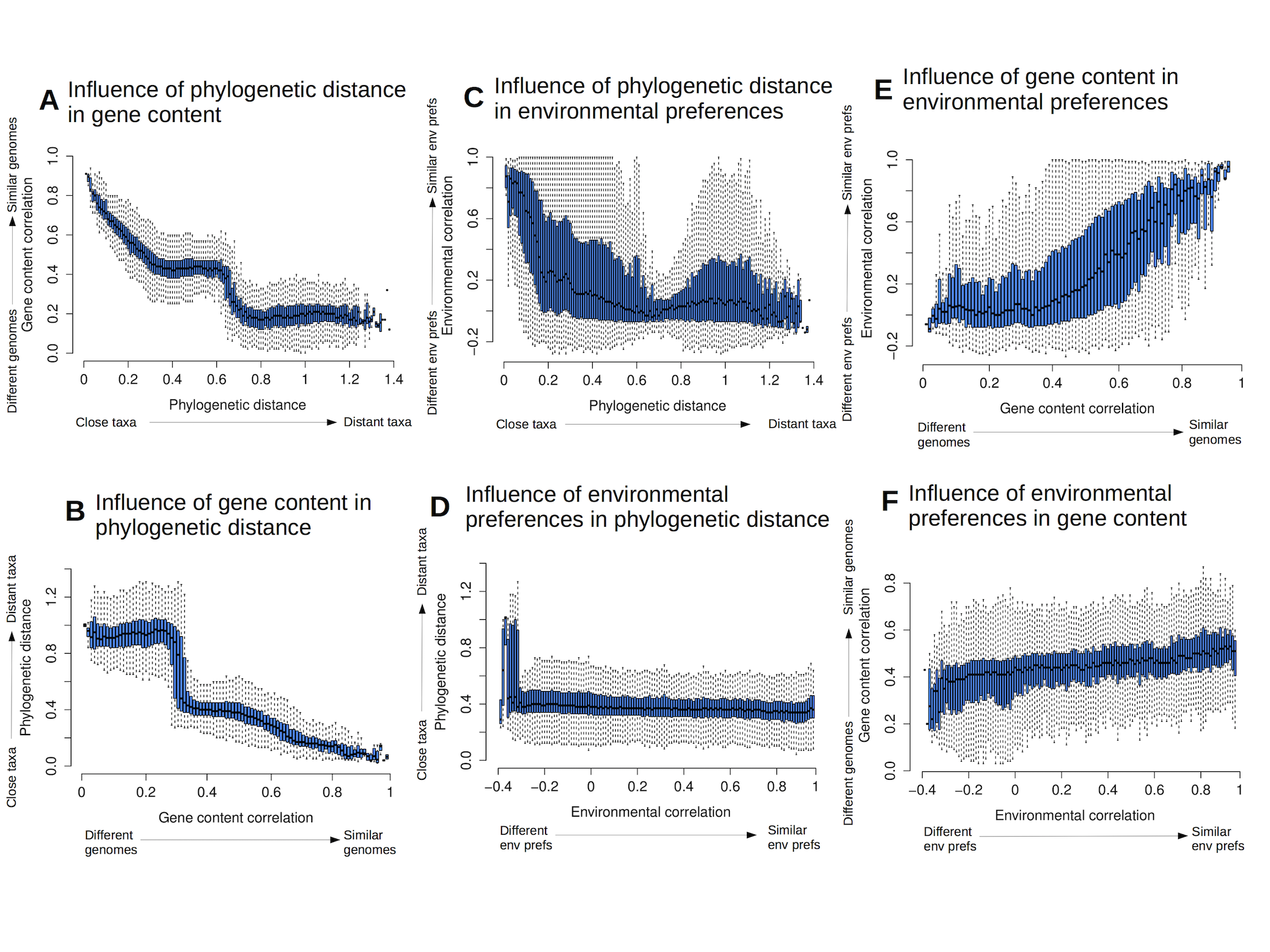

Supplement: Supplementary file 11 [file Image9.TIFF]

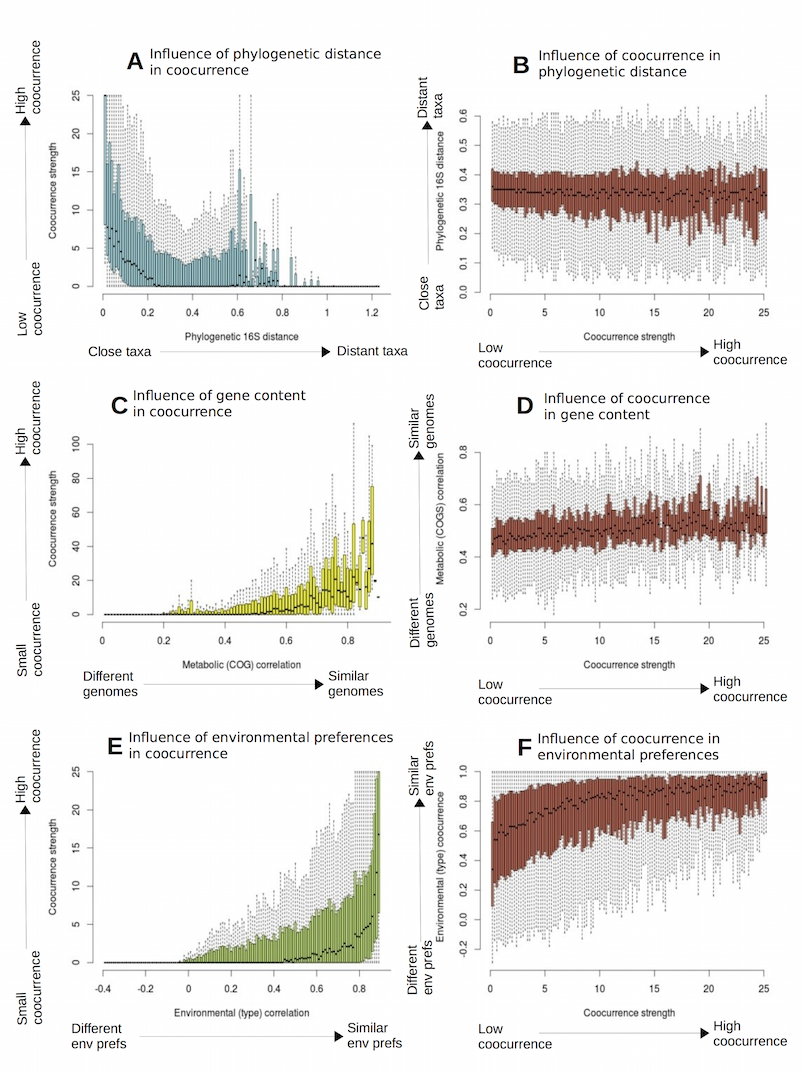

Supplement: Supplementary file 12 [file Image10.TIFF]

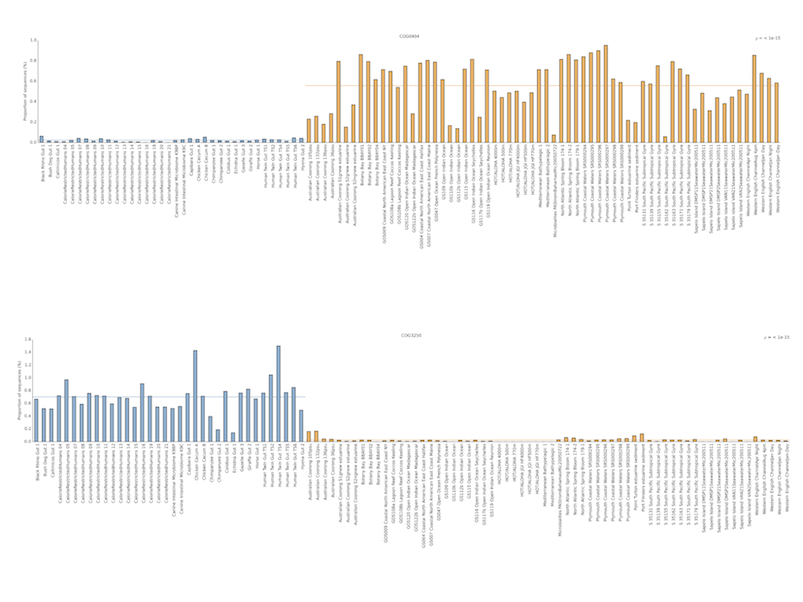

Supplement: Supplementary file 13 [file Image11.TIFF]
